# Effects of 5-Ion Beam Irradiation and Hindlimb Unloading on Metabolic Pathways in Plasma and Brain of Behaviorally Tested WAG/Rij Rats

**DOI:** 10.3389/fphys.2021.746509

**Published:** 2021-09-27

**Authors:** Jacob Raber, Sarah Holden, Reetesh Sudhakar, Reed Hall, Breanna Glaeser, Marek Lenarczyk, Kristen Rockwell, Natalie Nawarawong, Jennifer Sterrett, Ruby Perez, Scott William Leonard, Jeffrey Morré, Jaewoo Choi, Amy Kronenberg, Alexander Borg, Andy Kwok, Jan Frederik Stevens, Christopher M. Olsen, Jeffrey S. Willey, Gerd Bobe, John Baker

**Affiliations:** ^1^Department of Behavioral Neuroscience, Oregon Health and Science University, Portland, OR, United States; ^2^Department of Neurology, Psychiatry, and Radiation Medicine, Division of Neuroscience, ONPRC, Oregon Health and Science University, Portland, OR, United States; ^3^College of Pharmacy, Oregon State University, Corvallis, OR, United States; ^4^Department of Pharmacology and Toxicology, Neuroscience Center, Medical College of Wisconsin, Milwaukee, WI, United States; ^5^Radiation Biosciences Laboratory, Medical College of Wisconsin, Milwaukee, WI, United States; ^6^Department of Radiation Oncology, Wake Forest School of Medicine, Winston-Salem, NC, United States; ^7^Mass Spectrometry Core, Oregon State University, Corvallis, OR, United States; ^8^Linus Pauling Institute, Oregon State University, Corvallis, OR, United States; ^9^Biological Systems and Engineering Division, Lawrence Berkeley National Laboratory, Berkeley, CA, United States; ^10^Department of Animal Sciences, Oregon State University, Corvallis, OR, United States

**Keywords:** WAG/Rij/Cmcr, rats, simplified GCR simulation, space radiation, hindlimb unloading, open field, forced swim test, metabolomics

## Abstract

A limitation of simulated space radiation studies is that radiation exposure is not the only environmental challenge astronauts face during missions. Therefore, we characterized behavioral and cognitive performance of male WAG/Rij rats 3 months after sham-irradiation or total body irradiation with a simplified 5-ion mixed beam exposure in the absence or presence of simulated weightlessness using hindlimb unloading (HU) alone. Six months following behavioral and cognitive testing or 9 months following sham-irradiation or total body irradiation, plasma and brain tissues (hippocampus and cortex) were processed to determine whether the behavioral and cognitive effects were associated with long-term alterations in metabolic pathways in plasma and brain. Sham HU, but not irradiated HU, rats were impaired in spatial habituation learning. Rats irradiated with 1.5 Gy showed increased depressive-like behaviors. This was seen in the absence but not presence of HU. Thus, HU has differential effects in sham-irradiated and irradiated animals and specific behavioral measures are associated with plasma levels of distinct metabolites 6 months later. The combined effects of HU and radiation on metabolic pathways in plasma and brain illustrate the complex interaction of environmental stressors and highlights the importance of assessing these interactions.

## Introduction

The space environment consists of multiple charged particles that may impact brain function during and after exploratory missions. Various studies have reported effects of limited types of simulated space radiation on brain function in mice (Raber et al., 2015; Impey et al., 2016; Raber et al., 2016; Acharya et al., 2019; Raber et al., 2019; Torres et al., 2019) and rats (Rabin et al., 2015; Britten et al., 2017; Mange et al., 2018). Several of these studies considered behavioral and cognitive effects of single ions, but several considered “*ad hoc*” combinations of multiple ion beams delivered sequentially (Raber et al., 2019; Raber et al., 2020).

While much has been learned from simulated space radiation studies to date, a limitation is that radiation exposure is not the only environmental challenge astronauts face during missions. For example, microgravity is another challenge astronauts face during space missions. Therefore, models of simulated microgravity are being used in humans (Alessandri et al., 2012) and animal models (Ray et al., 2001; Morey-Holton and Globus, 2002). Microgravity alone has been suggested as a cause of brain dysfunction. For example, simulated microgravity affected metabolic pathways in the hippocampus (Sarkar et al., 2006). In addition, simulated microgravity during parabolic flights impaired the 3-dimensional visuospatial tuning and orientation of mice (Oman, 2007). Space radiation and microgravity might interact in how they affect biological outcome measures, including DNA damage (Moreno-Villanueva et al., 2017), other injury-related cellular pathways like oxidative stress and mitochondrial function (Yatagai et al., 2019), cardiovascular health (Patel, 2020), and bone function (Krause et al., 2017). Space radiation and microgravity might also interact in their effects on brain function.

NASA is actively planning exploratory missions where astronauts will be exposed to prolonged exposure to radiation and microgravity. The rat is accepted by NASA as a model organism to study the health effects of simulated galactic cosmic rays and microgravity. The WAG/Rij strain of rat is sensitive to radiogenic cardiovascular disease after exposure to low energy photons or mixed beams of high-energy charged particle radiation. Simulated space radiation and combined simulated microgravity and simulated space radiation, but not simulated microgravity by itself, induced vascular endothelial cell dysfunction (Delp et al., 2016). Prior to conducting exploratory missions, NASA needs to understand the impact of space radiation and microgravity on the central nervous system. The WAG/Rij rat is of interest in the context of long-term space missions, as this strain exhibits depression-like symptoms (Sarkisova and Kulikov, 2006; Sarkisova and Van Luijtelaar, 2011; Russo et al., 2014). These symptoms include: (1) decreased investigative activity in the open field test; (2) increased immobility in the forced swimming test; (3) decreased sucrose consumption and preference (anhedonia); (4) adopting passive strategies in stressful situations, helplessness, and submissiveness: and (5) inability to make choice and overcome obstacles. In addition, there are alterations in serotonin and dopamine levels in the brain, which are typical for depressed patients (Sarkisova and Van Luijtelaar, 2011). Depressive behavior, and mental health in general, is very pertinent to successful space missions (Kanas, 2016), especially longer deep space missions as those planned to Mars, and we reported increased depressive-like behavior of B6D2F1 mice 2 months following sequential radiation with protons (1 GeV, 60%, LET = 0.2 keV/μm), ^16^O (250 MeV/n, 20%, LET = 25 keV/μm), and ^28^Si (263 MeV/n, 20%, LET = 78 keV/μm) at a total dose of 0.5 Gy, a mission-relevant dose for exploratory class missions (Raber et al., 2019). Conducting studies of space radiation and simulated microgravity in the WAG/Rij rat allows comparisons to be made between the central nervous and cardiovascular systems.

In this study, we characterized behavioral and cognitive performance of male WAG/Rij/Cmcr rats 3 months after sham-irradiation or total body irradiation with a specially designed simplified galactic cosmic radiation simulator (simGCRsim) prescribed by NASA, comprised of five ions delivered at six discrete energies in rapid succession (0, 0.75 or 1.5 Gy; Simonsen et al., 2020). In addition, to assess the role of microgravity, we included experimental groups of simulated weightlessness using hindlimb unloading (HU) alone and HU in combination with simGCRsim exposure (1.5 Gy). Six months following behavioral and cognitive testing (9 months following sham-irradiation or total body irradiation), plasma and brain tissues (hippocampus and cortex) were processed to determine whether the behavioral and cognitive effects were associated with long-term alterations in metabolic pathways in plasma and brain.

## Materials and Methods

All methods were carried out in accordance with relevant guidelines and regulations and approved by the IACUC of BNL and MCW.

### Animals, Hindlimb Unloading and Radiation Exposures

**FIGURE 1:**
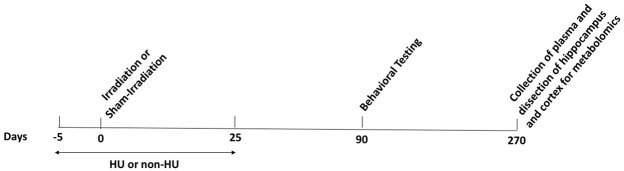
Timeline of the study. The animals received hindlimb unloading (HU) or not (non-HU) starting 5 days before sham-irradiated or irradiation. The HU condition continued 25 days after the sham-irradiation or irradiations. The animals were behaviorally and cognitively tested 90 days after sham-irradiation or irradiation. Collection of plasma and dissection of the hippocampus and cortex for metabolomics occurred 270 days after sham-irradiation or irradiation.

The outline of the study is illustrated in Figure 1. The opportunistic current study took advantage of the availability of sham-irradiated and irradiated WAG/Rij/Cmcr (WAG) rats part of a cardiovascular study in the Baker laboratory at MCW. Due to the longitudinal design of the cardiovascular study to assess late changes in cardiovascular function, the animals were euthanized 6 months following behavioral and cognitive testing and 9 months following sham-irradiation or total body irradiation. As one goal of the study was to compare alterations in metabolic pathways in plasma with those in brain, plasma, hippocampus, and cortex were processed for metabolomics at this time point as described below.

WAG male rats (*n* = 69), a substrain of WAG/Rij, separated from the colony at Netherlands Organization for Applied Scientific Research (TNO, Rijswijk, Netherlands), in about 1975, and maintained at Medical College of Wisconsin (MCW), were shipped to the Brookhaven National Laboratory (BNL), Upton, NY. After 2 weeks of acclimatization, and at 9 months of age, they were sham-irradiated or irradiated with a standardized, simplified 5-ion exposure prescribed by NASA and referred to as simGCRsim (consisting of rapid, sequential exposure to protons (1000 MeV/n, LET = 0.2 keV/μm, 35%), ^28^Si ions (600 MeV/n, LET = 50.4 keV/μm, 1%), ^4^He ions (250 MeV/n, LET = 1.6 keV/μm, 18%), ^16^O ions (350 MeV/n, LET = 16.9 keV/μm, 6%), ^56^Fe ions (600 MeV/n, LET = 173.8 keV/μm, 1%), and protons (250 MeV/n, LET = 0.4 keV/μm, 39%). Rats were exposed to 0, 0.75 Gy or 1.5 Gy) at the NASA Space Radiation Laboratory (NSRL) at BNL on May 7 or May 8, 2019. The 1.5 Gy cohort included experimental groups of simulated weightlessness using HU alone and HU in combination with radiation. The HU procedure was initiated 5 days prior to sham-irradiation or irradiation as described below. The rats remained in the HU conditions 25 days following sham-irradiation or irradiation. The animals were group housed besides the period during HU and operant set shifting. One week following the end of the HU period, the animals were shipped back from BNL to MCW where the behavioral testing was conducted and other endpoints were collected. The animals were maintained on a Teklad 8904 diet (Indianapolis, IN, United States) and fed *ad libitum* during this study, except for the period of the operant set shifting test described below. The animals were housed in a reverse light cycle room with lights off 0730−1930. All behavioral and cognitive testing was performed during the dark period. All animal procedures were consistent with ARRIVE guidelines and reviewed and approved by the Institutional Animal Care and Use Committee at BNL and MCW. All analyzes were performed blinded to treatment. The code was broken once the data were analyzed.

### Hindlimb Unloading

On Day 1, the rats were randomly grouped to serve as either full weight-bearing on all four limbs, or to be HU via tail suspension with body weight borne on the two front limbs. The HU procedure was a modified version of the one previously published (Wiley et al., 2016). Briefly, rats were lightly anesthetized with isoflurane (2.5–3.0%; 100% oxygen flow rate). While anesthetized, the tail was cleaned using 70% isopropyl alcohol. Benzoin tincture was applied to the lateral surfaces of the tail prior to the placement of strips (1 cm wide by 30 cm long) of adhesive medical traction tape (3M, St. Paul, MN, United States). The tape was placed approximately 1 cm from base of the tail, spanning distally for approximately the next 3/4ths the length of the tail. The free end of the tape was looped through a catch that was part of a custom-build plastic ball bearing swivel. After passing through the swivel, the tape was symmetrically adhered to the other lateral side of the tail in a similar spatial manner. Three pieces of 1/2-in micropore surgical tape (3M, St. Paul, MN, United States) were secured perpendicularly along the traction tape. A 1.56-in stainless oval wire carabiner clip (Nite Ize, Boulder, CO, United States) was attached through a hole on the top portion of the plastic swivel, with the other end of the carabiner clip attached to a zipper hook that slides along a perlon cord (STAS group, BA, Eindhoven, Netherlands). The cord was attached to a clothesline support separator spreader pulley (Blue Hawk, Gilbert, AZ, United States) that slides along a 5/16-in diameter round, solid steel rod (Hillman, Cincinnati, OH, United States). Once the attachment was complete, the zipper lock was adjusted along the perlon cord to lift the hind limbs fully off the substrate with a pitch angle of the thorax and abdomen 30° relative to the horizontal substrate plane. Rats quickly recovered from anesthesia and were monitored closely for the next 24 h. Rats maintained under standard gravitational conditions (non-HU) received isoflurane, but were not tail suspended. All HU and non-HU rats were further subgrouped as to receive one of the two radiation doses (0.75 Gy or 1.5 Gy) or sham-irradiation (0 Gy), and all animals were singly housed. Monitoring over the next 5 days was carefully performed to ensure the HU rats had access to food and water and could move smoothly across the cage, despite the hind limbs not touching the substrate. Wet rodent chow and gel packs were provided to ensure hydration. Corrugated cardboard bedding was supplied to permit burrowing for all rats. On Day 5, animals were transferred from HU or non-HU housing to a new plexiglass box (holder) drilled with air holes for radiation or sham-radiation exposure. HU animals remained suspended through the transferring process by removing the plastic ball bearing from the carabiner and relocating the animal to a plastic plexiglass holder (9.5″ × 4″ × 7.5″) where plastic bead chains were attached to the swivel. The new holders were placed into a rigid foam frame allowing six rats (in six holders) to be placed into the beamline simultaneously. The frame accommodated lengths of plastic bead-chains hanging from a top cross bar. One bead chain was looped through the hole in the plastic swivel, and the free end of the bead chain was affixed to itself using a plastic zip tie. The bead chain was then adjusted at the cross bar to ensure a 30° of the body of the relative to the plexiglass floor of the holder, with the hind limbs remaining off the floor. Each holder lid contained a cut-out section to permit closure with the tail extending upward toward the cross bar. This process was repeated five more times, such that a total six rats were HU and stacked (3 high; with two rats on the bottom, middle, and top levels) within the foam frame. Rats that did not receive HU were placed into similar plexiglass boxes drilled with airholes and stacked into rigid foam frames, but remained full weight-bearing. Their holders were smaller (8″ × 4″ × 4″), and were aligned in a matrix of three horizontal capsules and four vertical one for irradiation on the beam line (or sham-irradiation). Rats were then transported to the NSRL facility on each irradiation day for sham-irradiation or radiation exposure. All methods were carried out in accordance with relevant guidelines and regulations.

### Behavioral Studies

Behavioral studies were initiated 3 months following radiation or sham-irradiation, in August 2019.

### Performance in the Open Field in the Absence and Presence of Objects

Starting 3 months post-irradiation or sham-irradiation, exploratory behavior, measures of anxiety, and hippocampus-dependent spatial habituation learning were assessed in a black open field (90.49 cm × 90.49 cm × 45.72 cm) during two subsequent days. The rats were placed in the open field for 5 min per day. The animal cage was brought into the testing room. The rat was picked up gently placing thumb and fore finger behind the fore legs and wrapping the hand around the stomach. The researcher held the animal against the stomach while transporting the animal to or from the enclosure. The animal was placed in the middle of the open field. The researcher left the testing room during behavioral testing. Following testing of a rat, the enclosure was cleaned with a 70% isopropyl alcohol solution. Outcome measures analyzed using video tracking (Noldus Ethovision XT13, Wageningen, Netherlands) were the total distance moved and the percent time spent in the more anxiety-provoking center zone (45.25 × 45.25 cm).

On day 3, the rats were tested for in the open field containing objects based on the protocol using 3D printed objects as reported (Aguilar et al., 2018). The animals were first habituated to an open field one orange object and one cover of a 50 ml conical tube for 5 min. The objects were placed 45.72 cm from the top side of the enclosure and 30.48 cm from the left and right sides of the enclosure. The distance between the two objects was also 30.48 cm. Five minutes following the habituation trial (H), the rats were placed back in the enclosure containing two identical blue objects (squares) or two identical red objects (cylinders) for an acquisition trial. The objects were counterbalanced for this task. Half of the rats started with the blue squares and the other half started with the red cylinders. Sixty minutes following the acquisition trial (A), the rats were placed back in the enclosure with one of the familiar objects replaced with a novel red or blue object for a test trial (T). Outcome measures analyzed using video tracking were the total distance moved and percent time spent in the center zone (45.25 × 45.25 cm) containing the objects. In addition, the percent time the animals explored the novel and familiar objects in the test trial was analyzed by manual scoring of the digital videos and the discrimination index was calculated. The “discrimination index” was defined as [the time spent exploring the novel object (s)] – [time spent exploring the familiar object (s)]/(total time spent exploring both objects). The light intensity during open field and object recognition testing was 50 lux. A white noise generator (setting II) and overhead lights were used during the testing.

### Forced Swim Test

Following open field and object recognition testing, depressive-like behaviors of the rats were tested in the forced swim test. Rats were placed inside transparent round cylinders (91.44 cm high, diameter: 20.38 cm) filled 2/3 with water (28°C) to a designated level (∼60.69 cm) that allowed the tail, but not hind paws, to touch the bottom of the cylinder. A white noise generator (setting II) and overhead lights were used during the test. The rats were tested two at a time with an opaque separator placed between the two set ups. The rats were place in the cylinder for 6 min. Following removal from the water, they were dried off with paper towels, and monitored for 5 min in a warmed cage before being returned to their home cage. After 2–3 animals, the water was removed, and the cylinder was refilled with clean water. The outcome measure was the percentage of time the rats were immobile during the period of the test, as analyzed by manual scoring of the digital videos. The light intensity during the forced swim testing was 120 lux.

### Operant Set-Shifting and Reversal Tests

Finally, a subgroup of animals [*n* = 48 rats; four experimental groups: sham-irradiated, irradiated (0.75 or 1.5 Gy), and irradiated (1.5 Gy) plus HU] were tested for testing of operant set-shifting and reversal. Operant set-shifting and reversal were performed as described (muelbl et al., 2018) using methods adapted from Floresco et al. (2008). There were daily sessions. Rats were food restricted (∼85% free-feeding weight) beginning 3 days before the pre-training phase and continued throughout all operant testing. The day before pre-training, each rat was given 20 sucrose pellets that would be used as reinforcers during all training sessions.

The first pre-training phase involved a single lever press that resulted in a sucrose pellet reinforcer. Only one lever was presented in each session (either left or right, which was counterbalanced across groups). The criterion was met once 50 sucrose pellet reinforcers were given in a 30-minute session. Once criterion was reached, the rats were trained on the opposite lever with the same stipulations.

The second pre-training phase involved 90 distinct trials. In each trial, a random, single lever (left or right) was extended and available for a maximum of 10 s. Within that time window, a lever press would result in a sucrose pellet reinforcer, retraction of the lever, a house light shut off, and a 10 s time out period. The end of the time out period was signaled by the onset of the house light and a new lever exposure. If a lever was not pressed during the 10 s of exposure, the lever was retracted and counted as an omission. The 10 s time out period would still follow regardless, and the next trial would start. Rats had to complete this training for a minimum of four sessions. In order to pass on to the next phase, the rats needed to have fewer than 15 omissions in a session.

The last part of the pre-training phase was a solitary session to determine if a rat had developed a side bias during the previous tests. There were seven total trials in the session, which consisted of two phases. During the first phase, the house light was illuminated and both left and right levers were exposed. If either lever was pressed, a sucrose pellet reinforcer was presented, and the levers were retracted. The second phase involved in either a “correct” or “incorrect” lever response. At the beginning of this phase, both levers were once again presented, but only an opposite lever press compared to that of the first phase resulted in a sucrose pellet reinforcer. This was designated a “correct” response, and a new trial began. If the same lever was pressed in the second phase as the first, the result would be the absence of a reinforcer and a shut-down of the house light. This outcome was designated as “incorrect”. This second phase would be repeated until the “correct” response was completed. The biased side was determined by the first phase’s greatest number of lever presses on a given side.

The next testing phase was visual-cue discrimination. During this phase, the ability of the rats to detect a correct lever by selecting the lever with a visual cue light illuminated above it was assessed. There was a minimum requirement of 30 trials and a maximum of 150 trials in each session. The session either ended after 10 consecutive correct trials (excluding omissions) or after the 150th trial. These trials lasted 20 s. During the trials, both left and right levers were exposed, and one cue light was presented above the reinforced lever. The side of the cue light was randomly selected within each trial. If the rat pressed the lever below the cue light, the rat received the sucrose pellet reinforcer, levers were pulled back, the cue light turned off, and a “correct” lever response was recorded. An incorrect response was recorded if a rat pressed the lever opposite of the illuminated cue light. The response was followed by the cue light turning off and both levers being pulled back. A reinforcer was not provided in this instance. If a rat did not press a lever within the first 10 s of the trial, the levers were withdrawn, the cue light was turned off, and an omission was recorded. Following each scenario, a 10 s time out occurred before the start of the next trial. If the rats did not meet the criterion of 10 consecutive correct trials in one session, the sessions would be repeated on subsequent days until the criterion was met.

Once the criterion was met for the visual-cue discrimination test, the rats were tested for shift-to-response discrimination. In this phase, the rat was trained to select the “correct” lever based on a spatial rule rather than a visual cue rule. The visual cue light was independent of the correct lever, and the correct lever was either always the left or the right one for each trial. Pressing of the “correct” lever resulted in a sucrose pellet reinforcer. The selected “correct” lever was chosen by the software based on the Side Bias Determination test and was the least responded lever. The session started with 20 “reminder” trials that followed the outcomes of the previous visual cue discrimination test. All subsequent trials followed the new spatial rule of discounting the visual cue light and pressing of either the left or right lever. These reminder sessions allowed analyzing the 24-hour recall of the previous test to see if the learned rule was maintained. In addition, these reminder sessions provided a baseline performance of the visual-cue rule directly before the rule shift. There were 180 trials with the spatial cue rule. Thus, with the addition of the 20 “reminder” sessions, the rats received 200 total trials in this phase. Other than the reward deliveries, each trial’s presentation was duplicated from the visual-cue discrimination test. Within these 180 trials of learning the spatial rule, reaching a criterion of successful performance was defined as 10 consecutive correct trials. If the criterion was not reached in the session, the next day this training phase was repeated. There were three types of errors that could have occurred to prevent meeting the criterion: perseverative, regressive, or never reinforced related errors. A perseverative error occurred when the rat reached 75% of the required 10 correct responses within a block of 16 trials. A regressive error occurred when the rat reached the 75% of correct responses within the same block of 16 trials. A never reinforced error occurred when an incorrect response was not associated with the cue light.

After the shift-to-response discrimination test criterion was met, the rats were tested for response reversal the next day. Similar to the last phase, the session began with 20 “reminder” trials of the previous test phase. The following 180 trials followed that same spatial rule with the exception of the “correct” side. For example, if the “correct” side of the Shift-to-response test was the left side lever, the response reversal test’s “correct” lever would be the right-side lever. The same criterion of 10 consecutive correct trials was required as a positive response for this test phase as well. If the criterion was not met, this test phase would be repeated on the next day.

Finally, the rats were tested for progressive ratio test performance to gauge the motivation to acquire the sucrose pellet reinforcer. This test was phase occurred after the response reversal test. The “correct” lever of the response reversal test was the only lever exposed to the rat during this phase. For 1 h, the rats were able to press this lever, without constraints, on a progressive ratio schedule of reinforcement. Each ratio was calculated based on the formula described in Richardson and Roberts (1996), using *j* = 0.18. During this session, the house light remained on for its entirety and there were no cue lights.

### Metabolomics Analysis of Plasma, Hippocampus, and Cortex

Nine months following sham-irradiation or irradiation and 6 months after the behavioral and cognitive testing, in February 2020, sham-irradiated rats and rats irradiated at 1.5 Gy, in the absence and presence of HU, were quickly killed using a guillotine without anesthesia. The blood was collected in EDTA-containing tubes, centrifugated at 2,000 *g* for 10 min and the plasma supernatant collected and stored at −80°C. The brain was cut along the midline on ice in PBS and the whole hippocampus and whole cortex were dissected as described (Raber et al., 2002) and stored at −80°C. The tissues were homogenized in 300 μL (per 30 mg of tissues) of cold methanol: water (8:2, v/v). From 100 μl of plasma or hippocampal or cortical homogenates, metabolites were extracted. Untargeted metabolomics was performed as described (Kirkwood et al., 2013). Liquid chromatography (LC) was performed using a Shimadzu Nexera system with an Inertsil Phenyl-3 column (4.6 × 150 mm, 100 Å, 5 μm; GL Sciences, Rolling Hills Estates, CA, United States) coupled to a quadrupole time-of-flight mass spectrometer (Q-TOF) (AB SCIEX, Triple TOF 5600) operated in information-dependent MS/MS acquisition mode. Samples were ordered randomly and multiple quality control samples were included. QC samples were generated by pooling 10 μL aliquots from hippocampi or cortical tissues or plasma samples extracts and analyzed along with the samples. Samples were run in the positive and negative ion mode. In case metabolites were present in both ion modes, the mode with the higher peak value was selected for further analysis. The column temperature was held at 50°C and the samples were kept at 10°C. Samples from the cortex and hippocampus were analyzed as raw data and also analyzed after normalization to tissue weight. The metabolomics data were processed using Markerview (SCIEX, Framingham, MA, United States) and Peakview (SCIEX, Framingham, MA, United States) software for integrated pathway and statistical analyses. Identification of metabolites was based on mass error (<30 ppm) and MS/MS fragment ions. Metabolites were also confirmed using retention time, mass-to-charge (m/z) ratio, and comparison to authentic standards (±1 min) from an in-house library (IROA Technologies, Bolton, MA, United States), allowing for the streamlined identification of metabolites. LipidMaps (Welcome Trus, United Kingdom), METLIN (Scripps, La Jolla, CA, United States) and HMDB (University of Alberta, Edmonton, AB, Canada) databases were used for MS and MS/MS matching. Metaboanalyst pathway analysis (Montreal, Quebec, Canada) was performed as described by Xia and Wishart (2010) and Kirkwood et al. (2013). Metabolite peak area values in the plasma, cortex, and hippocampus were analyzed, without log transformation or Pareto scaling. Four distinct comparative analyses were performed: (1) effects of radiation in rats without HU; (2) effects of Radiation in rats with HU; (3) effects of HU in sham-irradiated rats; and (4) effects of HU in irradiated rats. Pathways were visualized using scatter plots (testing significant features) in Metaboanalyst, with the “global test” and “relative-betweenness centrality” as parameters for enrichment method and topological analysis, respectively.

### Statistical Analyses

For the cognitive and behavioral testing, all data are summarized as mean ± standard error of the mean (SEM). Behavioral and cognitive data were analyzing with SPSS v.25 software (IBM, Armonk, NY, United States). To analyze behavioral and cognitive performance after sham-irradiation or total body irradiation with a simplified 5-ion mixed beam exposure (0, 0.75, or 1.5 Gy), we performed analyses of variance (ANOVAs) with *post hoc* tests comparing to sham-irradiated animals when appropriate. To assess the role of microgravity, we performed ANOVAs including radiation (0 or 1.5 Gy) and HU condition (control or HU) as between group factors with repeated measures when appropriate. For some analyses, including comparing activity levels in the A and T trials when there was a radiation × test interaction, as indicated and appropriate, two-sided *t*-tests were used. For example, paired student *t*-tests were used to assess object preference in the object recognition test. We set statistical significance to *p* < 0.05. Greenhouse–Geisser corrections were used if sphericity was shown to be violated (Mauchly’s test).

Metabolomic profiles of plasma, hippocampus, and cortex were analyzed for the four comparisons described above. Of the three comparisons: (1) sham-radiation versus radiation (no HU); (2) HU versus no HU (no radiation); and (3) HU versus HU + radiation, we indicated pathways in red that were revealed in these three comparisons of plasma and the hippocampus and pathways in blue in which all three comparisons were revealed in one tissue (plasma or brain) and 2 of the 3 comparisons were revealed in the other tissue (plasma or brain). To identify potential plasma measures of radiation exposure or HU condition on behavioral or cognitive performance, we used regression analyses. We selected those metabolites that were most consistently included in the models (at least 10 times out of potentially 21 times). MetaboAnalyst software was used to generate impact plots. Graphs were generated using GraphPad software v.8.2.0 (La Jolla, CA, United States). As MetaboAnalyst is not ideal to analyze amino acid-related pathways, we analyzed them separately.

## Results

### Performance in the Open Field Without and With Objects

**FIGURE 2:**
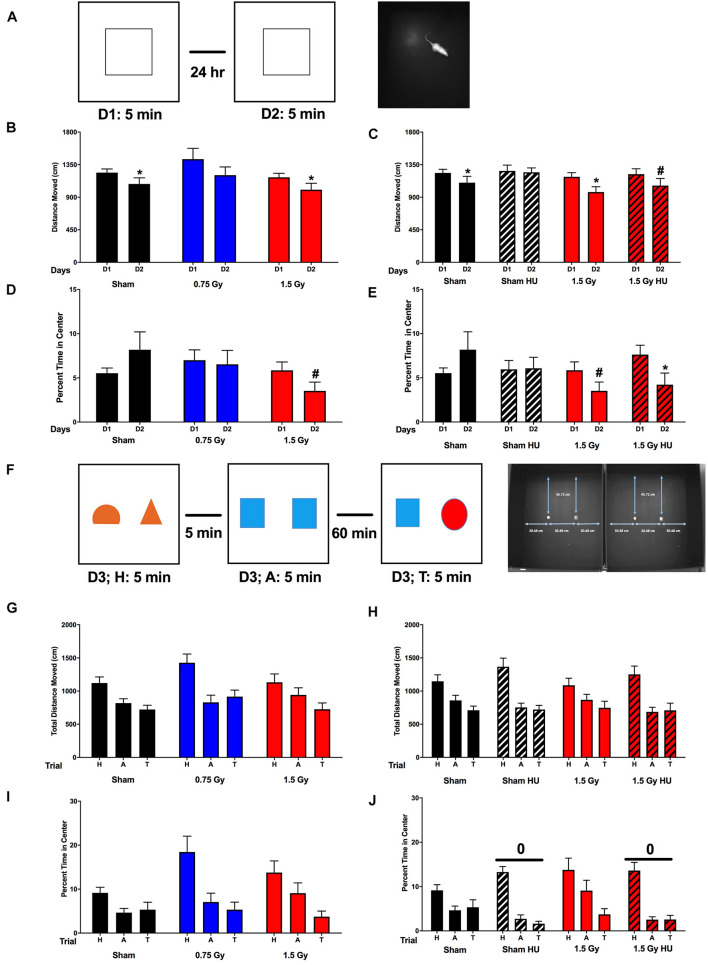
Performance in the open field in the absence and presence of objects. **(A)** The rats were tested for exploratory behavior in the open field on two subsequent days. The trials lasts 5 min and were 24 h apart. To assess measure of anxiety, the percent time spent in the more anxiety-provoking center of the open field was assessed as well. **(B)** Activity in the open field in sham-irradiated and irradiated animals. There was no main effect of radiation. However, sham-irradiated rats and rats irradiated with 1.5 Gy, but not those irradiated with 0.75 Gy, showed spatial habituation learning and moved less on day 2 than day 1. *p < 0.05 versus D1. **(C)** Exploratory activity in the open field in sham-irradiated and irradiated animals in the absence and presence of HU. There was no effect of HU or a radiation × HU interaction. However, sham-irradiated HU rats did not show spatial habituation learning and showed similar activity levels on both days, while there was a trend toward spatial habituation learning in rats that received both HU and irradiation. *p < 0.05 versus D1, ^#^*p* = 0.07 versus D1. **(D)** Measures of anxiety of sham-irradiated and irradiated animals in the open field. There was no main effect of radiation. However, there was a trend of rats irradiated with 1.5 Gy toward spending less time in the center on day 2 than day 1. ^#^*p* = 0.07. **(E)** There was no effect of HU or a radiation × HU interaction but there was a trend of HU irradiated rats toward spending less time in the center of the open field in day 2 than day 1. *p = 0.05, ^#^*p* = 0.07. **(F)** On the third day, the rats were tested in the open field containing objects. First they were habituated to objects in the open field for 5 min (H: habituation). After 5 min, they were tested in an open field containing two identical objects (A: acquisition). Sixty minutes later, they were tested in the open field containing one of the objects that was present in the acquisition test and one novel object. **(G)** Activity of sham-irradiated and irradiated animals in the open field containing objects. There was an effect of radiation [*F*(2,106) = 66.11, *p* < 0.0001] and a radiation × test interaction [*F*(6,106) = 2.606, *p* = 0.0214]. Activity levels were lower in the T than A trials in sham-irradiated animals and animals irradiated with 1.5 Gy. In contrast, in animals irradiated with 0.75 Gy, the activity levels in the A and T trials were comparable. **(H)** Activity of sham-irradiated and irradiated animals in the open field containing objects in the absence and presence of HU. There was a condition × test interaction [*F*(6,108) = 2.324, *p* = 0.0378]. Activity levels were lower in the T than A trials in sham-irradiated animals and animals irradiated with 1.5 Gy in the absence of HU, while sham-irradiated HU rats and irradiated HU rats showed comparable activity during the A and T trials. **(I)** Percent time spent in the center of sham-irradiated and irradiated animals. There was a radiation × trial interaction [*F*(4,84) = 3.935, *p* = 0.006]. Sham-irradiated animals and animals irradiated with 0.75 Gy spent a similar percent time in the center in the A and T trials. In contrast, animals irradiated with 1.5 Gy spent showed a trend toward spending less time in the center in the T than A trials. **(J)** Percent time spent in the center of sham-irradiated and irradiated animals in the absence and presence of HU. There was an effect of HU [*F*(2,108) = 6.214, *p* = 0.003] and a trend toward a trial × condition interaction [*F*(2,108) = 2.479, *p* = 0.089]. ^0^*p* = 0.003 versus non-HU groups. This interaction seemed driven by the sham-irradiated and irradiated HU rats pending less time in the center during the A and T trials than sham-irradiated and irradiated animals that had not received HU. OF, open field test; NO, novel object recognition test.

When activity levels were analyzed for two subsequent days in the open field (Figure 2A), there was no main effect of radiation (Figure 2C) or HU, or a radiation × HU interaction (Figure 2D). However, sham-irradiated rats and rats irradiated with 1.5 Gy of the simGCRsim beams in the absence of HU showed spatial habituation learning and moved significantly less on day 2 than day 1 (Figure 2C). Rats irradiated with 0.75 Gy simGCRsim alone showed the same pattern as the sham-irradiated and 1.5 Gy exposed cohorts, but did not reach statistical significance (Figure 2C). Sham-irradiated HU rats did not show spatial habituation at all and showed similar activity levels on both days, while there was a modest amount of spatial habituation learning in rats that received both HU and 1.5 Gy of simGCRsim but this did not reach statistical significance (Figure 2D). Next, we assessed time spent in the more anxiety-provoking center of the open field. There was no main effect of radiation (Figure 2E) or HU, or a radiation × HU interaction (Figure 2F). However, there was a trend of rats irradiated with 1.5 Gy toward spending less time in the center on day 2 than day 1 (*p* = 0.07, Figure 2E). This pattern was not seen in sham-irradiated rats nor was it seen in rats irradiated with 0.75 Gy (Figure 2E) and sham-irradiated HU rats (Figure 2F) but was seen in rats that received both radiation and HU (*p* = 0.05, Figure 2F).

Next, the performance in the open field containing objects was assessed (Figure 2B). There was an effect of radiation [*F*(2,106) = 66.11, *p* < 0.0001] and a radiation × test interaction [*F*(6,106) = 2.606, *p* = 0.0214; Figure 2G). The activity was lower in the T than A trials in sham-irradiated animals (*t* = 2.095, *p* = 0.0484, 2-tailed) without HU and there was a trend toward the activity being lower in the T than A trials in rats irradiated with 1.5 Gy of simGCRsim alone (*t* = 1.948, *p* = 0.0774, 2-tailed). In contrast, in animals irradiated with 0.75 Gy only, the activity levels in the A and T trials were comparable [Figure 2G, (*t* = 1.093, *p* = 0.302, 2-tailed)]. When we assessed the effects of HU in the presence and absence of radiation, there was a condition × test interaction [*F*(6,108) = 2.324, *p* = 0.0378, Figure 2H]. While activity levels were lower in the T than A trials in sham-irradiated and irradiated non-HU rats as described above, activity levels were comparable during the A and T trials in sham-irradiated (*t* = 0.1006, *p* = 0.9217, 2-tailed) and irradiated (*t* = 0.2439, *p* = 0.8118, 2-tailed) HU rats showed.

When the percentage of time spent in the center in the presence of the objects during the H, A, and T trials was analyzed, there was a radiation × trial interaction [*F*(4,84) = 3.935, *p* = 0.006]. Sham-irradiated animals (*t* = 0.4093, *p* = 0.6875, 2-tailed) and animals irradiated with 0.75 Gy simGCRsim (*t* = 1.469, *p* = 0.1800, 2-tailed) spent a similar percent time in the center in the A and T trials, while animals irradiated with 1.5 Gy simGCRsim showed a trend toward less time in the center in the T than A trials [Figure 2I, (*t* = 1.906, *p* = 0.0931, 2-tailed)]. When the effect of HU in the absence and presence of radiation was assessed, there was an effect of HU [*F*(2,108) = 6.214, *p* = 0.003] and a trend toward a trial × condition interaction [*F*(2,108) = 2.479, *p* = 0.089; Figure 2J]. Next, we analyzed the percent time spent with the familiar and novel objects and calculated the discrimination index. There was no preference for exploring the novel object in any group or group difference in the discrimination index (Supplementary Figure 1). This result might be related to the fact that most animals spent little time in the center and some animals did not explore the objects at all in the T trial.

### Depressive-Like Behavior in the Forced Swim Test

**FIGURE 3:**
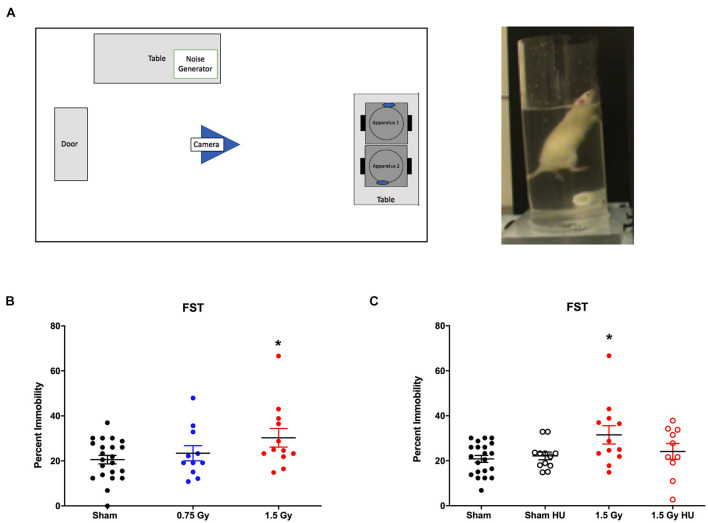
**(A)** Depressive-like behavior of sham-irradiated and irradiated animals in the forced swim test. Rats irradiated with 1.5 Gy spent more time immobile than sham-irradiated rats. *p < 0.05 versus sham-irradiated rats. **(B,C)** Depressive-like behavior of sham-irradiated and irradiated animals in the absence and presence of HU in the forced swim test. There was a radiation × HU interaction [*F*(1,50) = 6.591, *p* = 0.013]. Percent immobility was similar in rats that received only HU or HU and radiation exposure to that seen in sham-irradiated rats.

There was a trend toward an effect of radiation on depressive-like behavior, analyzed as percent immobility in the forced swim test [*F*(1,50) = 3.248, *p* = 0.078]. In rats without HU, those irradiated with 1.5 Gy simGCRsim spent more time immobile than sham-irradiated rats (*p* < 0.05, Figures 3A,B). There was a radiation × HU interaction [*F*(1,50) = 6.591, *p* = 0.013, Figure 3B]. Percent immobility was similar in rats that received only HU or HU and radiation exposure compared to that seen in sham-irradiated rats (Figure 3B). There was no additional effect of irradiation on depressive-like behavior in the HU rats as compared with the rats given HU only.

### Performance in the Operant Set-Shifting and Reversal Tests

There were no group differences in body weights before operant set-shifting and reversal testing (Supplementary Figure 2A). During visual cue learning (errors to criterion, Supplementary Figure 2B), and 24 h recall (Supplementary Figure 2C), there were no effects of radiation or HU. Similarly, there were no effects of radiation or HU during set shift errors (errors to criterion, Supplementary Figure 2D) or 24 h recall (Supplementary Figure 2E). Set shifting was not impaired in any group. There were no group differences in perseverative (Supplementary Figure 2F), regressive (Supplementary Figure 2G), or never reinforced errors (Supplementary Figure 2H). Reversal learning was also not different among the groups. There were no group differences in reversal errors (errors to criterion, Supplementary Figure 3A), perseverative (Supplementary Figure 3B), regressive reversal errors (Supplementary Figure 3C), in motivation to obtain food (Supplementary Figure 3D), in the number of sessions to criterion for single lever trainings (Supplementary Figure 4A), in the number of sessions to criterion for retract lever training (Supplementary Figure 4B), in the total number of pre-training sessions to reach criterion (Supplementary Figure 4C), or in cumulative active lever presses for the duration of the progressive ratio session (Supplementary Figure 5).

### Metabolomics Analysis of Plasma, Hippocampus, and Cortex

Nine months following sham-irradiation of simGCRsim irradiation and 6 months after the behavioral and cognitive testing, sham-irradiated rats and rats exposed to 1.5 Gy, in the absence and presence of HU, were quickly killed using a guillotine without anesthesia to prevent effects of the anesthesia on the metabolomics results. There were no group differences in body weights prior to euthanasia (Supplementary Figure 6). To assess alterations in metabolic pathways, four distinct comparative analyses were performed: (1) effects of radiation in rats without HU; (2) effects of Radiation in rats with HU; (3) effects of HU in sham-irradiated rats; and (4) effects of HU in irradiated rats.

**FIGURE 4:**
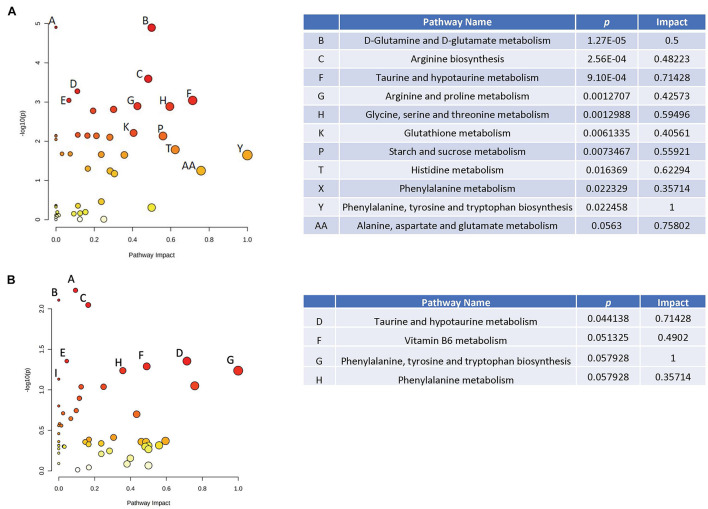
Metabolic pathways affected by radiation in the absence of HU in the plasma **(A)** and hippocampus **(B)**. When effects of radiation were analyzed in plasma in the absence of HU, several pathways were revealed **(A)**, including glutamine and glutamate metabolism, arginine biosynthesis, arginine and proline metabolism, taurine and hypotaurine metabolism, and glycine, serine, and threonine metabolism. Of those pathways, the taurine and hypotaurine metabolism and phenylalanine pathways were also affected in the hippocampus **(B)**. The metabolome view figures contain all the matched pathways (the metabolome) arranged by *p* values (generated as part of the pathway enrichment analysis) on the *Y*-axis, and pathway impact values (generated as part of the pathway topology analysis) on the *X*-axis. The node color is based on its *p* value and the node radius is determined based on their pathway impact values.

When effects of radiation were analyzed in plasma in the absence of HU, several pathways were revealed (Figure 4A), including glutamine and glutamate metabolism, arginine biosynthesis, arginine and proline metabolism, taurine and hypotaurine metabolism, and glycine, serine, and threonine metabolism. Of those pathways, the taurine and hypotaurine metabolism and phenylalanine pathways were also affected in the hippocampus (Figure 4B). The metabolome view figures contain all the matched pathways (the metabolome) arranged by *p* values (generated as part of the pathway enrichment analysis) on the *Y*-axis, and pathway impact values (generated as part of the pathway topology analysis) on the *X*-axis. The node color is based on its *p* value and the node radius is determined based on their pathway impact values.

**FIGURE 5:**
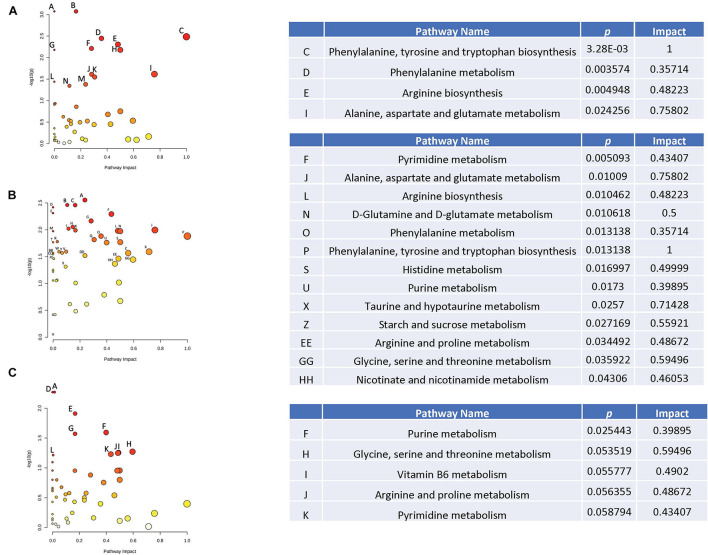
Metabolic pathways affected by radiation in the presence of HU in the plasma **(A)**, hippocampus **(B)**, and cortex **(C)**. When effects of radiation were analyzed in plasma in the presence of HU, the phenylalanine was affected **(A)** and was also affected in the hippocampus **(B)**. In the presence of HU, the arginine pathway was affected in plasma and also affected in the hippocampus **(B)** and cortex **(C)**. The taurine and hypotaurine pathway was affected in plasma **(A)** and the hippocampus **(B)**.

When effects of radiation were analyzed in plasma in the presence of HU, the phenylalanine was affected (Figure 5A) and was also affected in the hippocampus (Figure 5B). In addition, in the presence of HU, the arginine pathway was affected in plasma and also affected in the hippocampus (Figure 5B) and cortex (Figure 5C). The taurine and hypotaurine pathway was affected in plasma (Figure 5A) and the hippocampus (Figure 5B).

**FIGURE 6:**
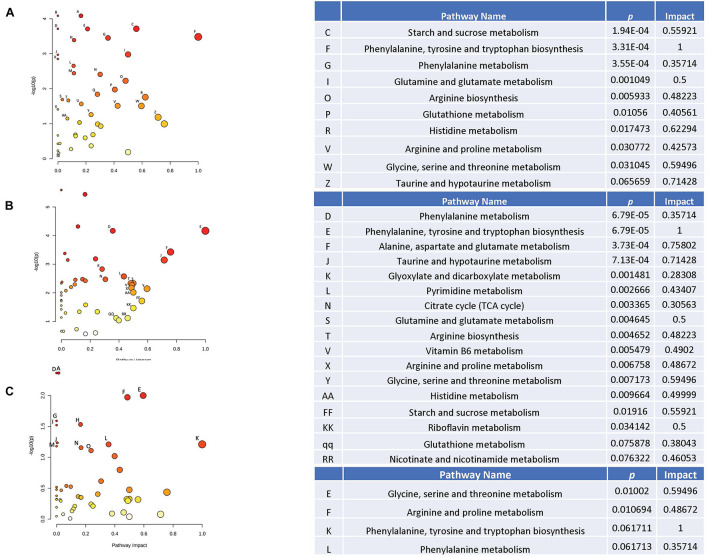
Metabolic pathways affected by HU in the plasma **(A)** and cortex **(B)** of sham-irradiated animals. Of the pathways affected by HU in plasma in sham-irradiated animals **(A)**, several were also affected in the hippocampus **(B)**, including the starch and sucrose, phenylalanine, glutamine and glutamate, glutathione, histidine, arginine and proline, glycine, serine, and threonine, and taurine, and hypotaurine pathways. The glycine, arginine, and phenylalanine pathway was affected by HU in the plasma **(A)** and also affected in the cortex **(C)**.

Next, effects of HU were analyzed in sham-irradiated animals. Of the pathways affected by HU in plasma (Figure 6A), several were also affected in the hippocampus (Figure 6B), including the starch and sucrose, phenylalanine, glutamine and glutamate, glutathione, histidine, arginine and proline, glycine, serine, and threonine, and taurine and hypotaurine pathways. The glycine, arginine, and phenylalanine pathway was affected by HU in the plasma (Figure 6A) and also affected in the cortex (Figure 6C).

**FIGURE 7:**
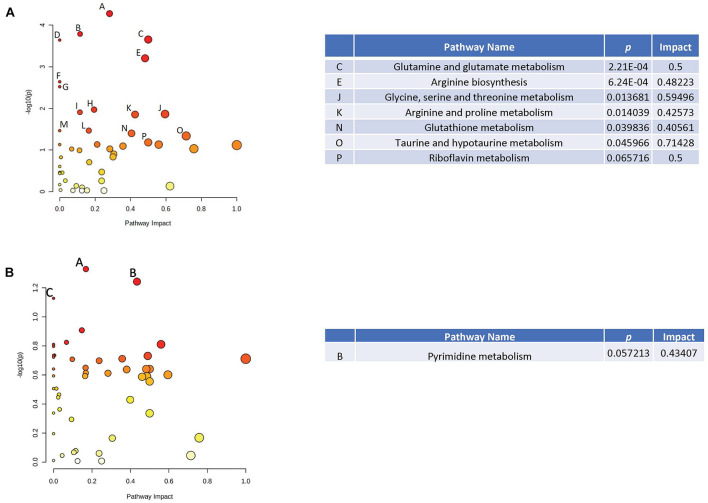
Metabolic pathways affected by HU in the plasma **(A)** and cortex **(B)** of irradiated animals. The effects of HU on metabolic pathways in plasma in irradiated animals **(A)** were less pronounced compared to the effects of HU alone on metabolic pathways in plasma of sham-irradiated animals (Figure 6A). None of the pathways affected by HU in plasma of irradiated animals were affected in the hippocampus or cortex. In the hippocampus, there were no pathways affected by HU and in the cortex only the pyrimidine pathway was affected by HU **(B)**.

Finally, we analyzed effects of HU in plasma of irradiated animals (Figure 7A). The effects of HU on metabolic pathways in plasma in irradiated animals (Figure 7A) were less pronounced compared to the effects of HU alone on metabolic pathways in plasma of sham-irradiated animals (Figure 6A). In addition, none of the pathways affected by HU in plasma of irradiated animals were affected in the hippocampus or cortex. In the hippocampus, there were no pathways affected by HU and in the cortex only the pyrimidine pathway was affected by HU (Figure 7B). The metabolites levels in the four groups in the affected pathways are illustrated in the plasma and cortex (Supplementary Figure 7) and hippocampus (Supplementary Figure 8).

Based on three comparisons (1) sham-radiation versus radiation (no HU); (2) HU versus no HU (no radiation); and (3) HU versus radiation + HU, affected pathways were revealed in these three comparisons in plasma and the hippocampus (indicated in bold and red in Table 1). In addition, based on these three comparisons, pathways were revealed in one tissue (plasma or brain) and based on 2 of the 3 comparisons in the other tissue (plasma or brain) (indicated in bold and blue in Table 1).

**TABLE 1:**
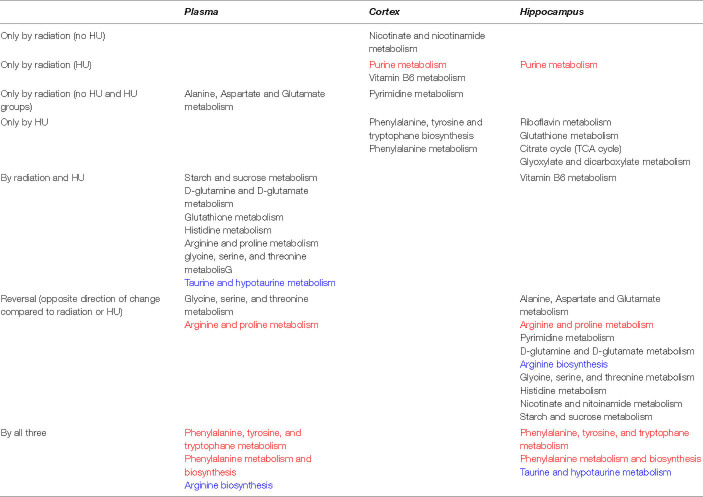
Pathways affected in plasma, hippocampus, and cortex based on three comparisons: (1) sham-radiation versus radiation (no HU); (2) Hindlimb unloading (HU) versus no HU (no radiation); and (3) HU versus radiation + HU, were revealed in these three comparisons^1^.

*^1^Red; pathways affected in plasma and the hippocampus.*

*Blue; one tissue (plasma or brain) reveals all 3 comparisons; other tissue (plasma or brain) reveals 2 out 3 comparisons.*

We identified 127 metabolites in the hippocampus. Radiation decreased metabolite levels overall. The main effect of radiation was significant for 33 metabolites (all higher with radiation) and the effect of radiation in rats without HU was significant for 10 metabolites (all lower with radiation). The effect of HU differed between un-radiated and irradiated rats. In sham-irradiated rats, the effect of HU was significant for 66 metabolites (all lower with HU). In irradiated rats, the effect of HU was significant for only 2 metabolites. Overall, the interaction between radiation and HU was significant for 50 metabolites. The lowest values were observed for HU sham-irradiated rats. For 33 metabolites, HU sham-irradiated rats had significantly lower values than each of the three other treatment groups.

We also identified 127 metabolites in the cortex. Compared to the hippocampus, the effects on metabolite levels in the cortex were small. The main effect of radiation was significant for 7 metabolites (4 higher with radiation) and the effect of radiation for rats without HU was significant for 9 metabolites (8 higher with radiation). The interaction between radiation and HU was significant for 20 metabolites compared with 7 metabolites for the main effect of radiation and 4 metabolites for the main effect of HU. Fourteen of the 20 significant metabolites were higher in sham-irradiated HU rats than the radiated HU rats or sham-irradiated rats without HU.

We identified 119 metabolites in the plasma. The effects in plasma were smaller than those in the hippocampus. The main effect of radiation was significant for 18 metabolites (8 higher with radiation) and the effect of radiation in rats without HU was significant for 41 metabolites (5 higher with radiation). The interaction between radiation and HU was significant for 37 metabolites compared with 18 metabolites for the main effect of radiation and 23 metabolites for the main effect of HU. For 10 metabolites, the sham-irradiated rats without HU had significantly different values than each of the three other treatment groups (7 higher and 3 lower in the sham-irradiated rats without HU).

To identify potential plasma measures of simGCRsim exposure or the HU condition on behavioral or cognitive performance, regression analyses were performed and those metabolites were that were most consistently included in the models (at least 10 times out of potentially 21 times) (Table 2).

**TABLE 2:**
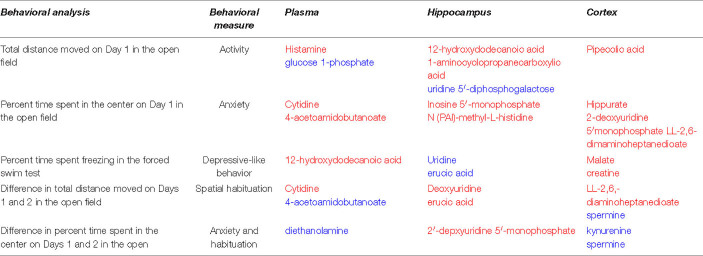
Relationship between behavioral measures and plasma metabolites revealed in at least 10 out of 21 comparisons^1^.

*^1^Red: decrease with change; blue: increase with change.*

Activity in day 1 in the open field was positively related to plasma glucose 1-phosphate levels and negatively related to plasma histamine levels. Activity in the open field was positively related to uridine 5′-diphosphogalactose levels and negatively to 12-hydroxydodecanoic acid in the hippocampus and negatively related to pipecolic acid levels in the cortex.

Measures of anxiety in the open field (percent time spent in the center of the open field on day 1) were negatively related to plasma levels of cytidine and 4-acetamidobutanoate levels in plasma, inosine 5′-monophosphate and N(PAI)-methyl-L-histidine in the hippocampus, and hippurate, 2′-deoxyuridine 5′-monophosphate, and 2,6-diaminoheptanedioate in the cortex.

Depressive-like behavior in the forced swim test (percent immobility) was negatively related to 12-hydroxydodecanoic acid levels in the plasma, positively related to uridine and erucic acid in the hippocampus, and negatively related to malate and creatinine levels in the cortex.

Spatial habituation (the difference in activity levels between days 1 and 2 in the open field) was positively related to 4-acetamidobutanoate and negatively related to cytidine levels in the plasma, negatively related to deoxyuridine and erucic acid in the hippocampus, positively related to spermine levels in the cortex and negatively related to 2,6-diaminoheptanedioate in the cortex. The negative relationship between spatial habituation and plasma cytidine levels is illustrated in Supplementary Figure 9. As 4-acetamidobutanoate is the immediate acetylated metabolite of the inhibitory neurotransmitter gamma aminobutyric acid (GABA) and a product of arginine and proline metabolism (Wahli and Guillou, 2021), this suggests a role for GABA neurotransmission in hippocampus-dependent spatial habituation learning. Based on this results, we compared GABA, glutamate, and GABA/glutamate ratios in the plasma, hippocampus, and cortex. In plasma, glutamate levels and the GABA/glutamate ratio were lower in rats following only radiation, only HU, or radiation and HU than sham-irradiated rats while there were no group differences in GABA levels (Table 3). In the hippocampus, GABA and glutamate levels in rats following only radiation or only HU, but not following radiation and HU, were lower than those in sham-irradiated rats, without a change in the GABA/glutamate ratio (Table 3). In the cortex, GABA levels were higher in rats following only HU and lower in rats following radiation and HU compared to those in sham-irradiated rats (Table 3). These data suggest that plasma glutamate levels might be a valuable measure of response to radiation, HU, or radiation and HU and that the hippocampus and cortex are able to preserve the GABA/glutamate ratio following those challenges.

**TABLE 3:** Gamma aminobutyric acid (GABA), glutamate, and GABA/glutamate ratios in the plasma, hippocampus, and cortex.

	Sham	Radiation only	HU only	Radiation + HU	SEM
***Cortex***					
GABA	**863999^ab^**	891388^ab^	**1077176^a^**	**799890^b^**	79531
Glutamate	10097241	10360432	10100411	9432767	484740
GABA/Glutamate	11.99	10.38	11.99	12.13	0.78
***Hippocampus***					
GABA	1000721^a^	**792764^b^**	**942366^ab^**	1057142^a^	69669
Glutamate	9493217^a^	**8472592^b^**	**9274650^ab^**	9833167^a^	328151
GABA/Glutamate	9.66	9.96	9.8	10.83	0.47
***Plasma***					
GABA	7892	7533	7760	8825	592
Glutamate	**352719^a^**	**266306^b^**	**269503^b^**	**267748^b^**	20271
GAB/Glutamate	**47.74^a^**	**35.33^b^**	**36.47^b^**	**30.84^b^**	2.89

*LS means in the same row with different superscripts differ at *p* < 0.05. The values in bold indicate the comparison groups.*

A combined measure of anxiety and habituation (the difference in percent time spent in the center of the open field on days 1 and 2) was positively related to diethanolamine levels in the plasma, negatively related to 2′-deoxyuridine 5′-monophosphate in the hippocampus, and positively related to kynurenine and spermine levels in the cortex.

These data show that specific behavioral measures several months after exposure are associated with plasma levels of distinct metabolites measured at a later time point.

## Discussion

In this study, we characterized behavioral and cognitive performance of 9-month-old male WAG/Rij/Cmcr rats after sham-irradiation or total body irradiation with a simplified GCR simulator (simGCRsim) spectrum prescribed by NASA, in the absence and presence of HU. The simGCRsim beam set was designed to broadly represent the distribution of primary and secondary ions produced behind shielding in a spacecraft or habitat, which is where crew members will spend the majority of their time on extended exploratory class missions (Simonsen et al., 2020). Astronauts will be exposed to the galactic cosmic radiation throughout a mission along with other stressors, of which microgravity is one of the most important. Sham HU, but not irradiated HU, rats were impaired in spatial habituation learning. Rats irradiated with 1.5 Gy of simGCRsim showed increased depressive-like behaviors. This was seen in the absence but not presence of HU. Both spatial habituation learning and depressive-like behaviors are mission relevant outcome measures.

Hindlimb unloading has differential effects in sham-irradiated and irradiated animals. In rats without HU, some commonalities were found in the metabolomics analysis at the terminus of the present studies. Taurine and hypotaurine metabolism and phenylalanine pathways affected by radiation in plasma were also affected by radiation in the hippocampus. In rats with HU, phenylalanine pathways affected by radiation in plasma were also altered in the hippocampus. In sham-irradiated rats, starch and sucrose, phenylalanine, glutamine and glutamate, glutathione, histidine, arginine and proline, glycine, serine, and threonine, and taurine and hypotaurine pathways affected by HU in plasma also affected in the hippocampus. In addition, in sham-irradiated rats, glycine, arginine, and phenylalanine pathways affected by HU in plasma also affected in the cortex. These data show that phenylalanine, tyrosine, and tryptophan metabolism and phenylalanine metabolism and biosynthesis are very strong pathway changes revealed by the tissue comparisons. Arginine biosynthesis and taurine and hypotaurine metabolism are less strong pathway changes revealed by these comparisons. Thus, specific behavioral measures are associated with plasma levels of distinct metabolites 6 months after behavioral testing, suggesting that it will be possible to develop stable plasma measures of the radiation response, HU, and of behavioral performance. As 4-acetamidobutanoate is the immediate metabolite of GABA, the positive relationship between plasma 4-acetamidobutanoate levels and spatial habituation learning in the open field suggest a role of GABA neurotransmission in this hippocampus-dependent cognitive function. Consistent with this notion, GABA is important in olfactory memory (Boumghar et al., 2012) and synaptic plasticity in the hippocampus (Whissel et al., 2016). The direction of change following radiation might depend on the type of radiation, brain region, and measure of GABA function assessed. Cortical GABA levels are reduced following combined irradiation with gamma rays and ^12^C ions in Wistar rats (Kokhan et al., 2019), hypothalamic GABA levels are reduced following whole brain gamma irradiation while the GABA levels in the prefrontal cortex are not affected (Franco-Perez et al., 2020), but hippocampal GABA release is increased following proton irradiation (Lee et al., 2017).

The combined effects of HU and radiation on metabolic pathways in plasma and brain illustrates the complex interaction of environmental stressors and highlights the importance of assessing these interactions. Similar to what is seen in animals exposed to both radiation and traumatic brain injury (Allen et al., 2012, 2014), the time interval between the two environmental challenges might be important in modulating their interaction. It is conceivable that when given 5 days prior to radiation exposure as in the current study, HU might serve as preconditioning event triggering compensatory changes that might mediate some protection against a subsequent radiation challenge.

Compared to the cortex, metabolic pathways were in general more affected by radiation and HU in the hippocampus and plasma. Consistent with this pattern, while behavioral and cognitive measures that involve hippocampal function, like spatial habituation learning in the open field was affected by HU and depressive-like behavior in the forced swim test was affected by radiation, frontal-cortex-dependent operant tasks were not (muelbl et al., 2018). This does not mean that the cortex might not be affected by HU or radiation. In a blast model of traumatic brain injury, which causes bilateral damage in the medial prefrontal cortex, visual cue discrimination, but not set shifting or delayed matching to sample (mPFC-dependent tasks) were affected. Specifically, there were more errors during acquisition of a cue discrimination task and only subtle differences in cocaine-primed reinstatement 3, 4 months after blast injury (muelbl et al., 2018), although oxycodone seeking following self-administration was elevated in blast treated rats (Nawarawong et al., 2019). We recognize that a limitation of the current study design is that the whole cortex was processed for metabolomics. It is conceivable that anatomical specific effects of radiation and HU on metabolic pathways in cortical subregions might have contributed that in general more pathways were revealed to be affected in the hippocampus and cortex.

In the current study, plasma, hippocampal, and cortical tissues were analyzed 6 months after behavioral and cognitive testing 9 months after radiation and/or HU. The metabolic pathways affected by radiation and HU and the relationships between the behavioral and cognitive measures and individual metabolite levels in plasma, hippocampus, and cortex, suggest that it will be feasible to develop stable long-term biomarkers of the radiation response, response to HU, and of behavioral and cognitive performance. Several pathways affected by radiation or HU in plasma were also affected in hippocampus and/or cortex. These data suggest that analysis of peripheral measures might be used to reflect alterations in measures in brain. This does not mean that the measures in plasma and brain are necessarily the same one. The relationships of specific behavioral and cognitive measures with distinct metabolites in plasma, hippocampus, and cortex, illustrates the complexity involved. Nevertheless, these results are very encouraging and modulating the levels of those plasma metabolites might be a valuable approach to improve behavioral and cognitive function during and following space missions. We recognize that a limitation of the current study is that alterations in metabolic pathways in plasma were not assessed shortly after behavioral testing. Therefore, we do not know the time kinetics and stability of the pathway alterations revealed 6 months after behavioral testing. Future efforts are warranted to assess the time kinetics and stability of metabolic changes in plasma following behavioral testing after radiation and HU exposure.

The version of the object recognition test we used involves a trial in which animals are first habituated to objects prior to the acquisition testing (Aguilar et al., 2018). This habituation trial in included to enhance exploratory behavior with the objects during subsequent trials. However, a concern is that animals typically spent less time exploring objects in subsequent trials (Benice et al., 2006; Acevedo et al., 2007). The distance moved and time spent in the center of the open field that contained the objects during the habituation, acquisition, and test trials are consistent with this scenario. So, the inclusion of a habituation trial with objects might have contributed to a reduction in time that the animals spent exploring the objects in the acquisition and test trials. This in turn might have contributed to the lack of preference for exploring the novel object in any of the experimental groups. Although rats showed a robust preference in the object recognition test, using the same 3D printed objects, including the same colors, in Aguilar et al. (2018) study, the fact that rats do not see red might have contributed to the lack of a preference for the novel object in our study as well. The lack of a preference for exploring the novel object in sham-irradiated non-HU animals preclude from drawing conclusions on potential effects of HU and radiation on object recognition.

In sham-irradiated animals, HU had detrimental effects on hippocampus-dependent spatial habituation learning and affected metabolic pathways in plasma, hippocampus, and cortex as well. As HU did not affect prefrontal cortex-dependent cognitive testing, these data suggest that the hippocampus might be especially susceptible to effects of HU. These data are consistent with effects of simulated microgravity on metabolic pathways in the hippocampus (Sarkar et al., 2006) and detrimental effects of simulated microgravity on 3-dimensional visuospatial tuning and orientation of mice (Oman, 2007). As behavioral testing started 3 months following HU and plasma and brain tissues were analyzed 8 months following HU, these results indicate that HU has long-term effects on the brain. We recognize that detrimental effects of charged particle exposures involving single ions found in the GCR on prefrontal cortex-dependent operant conditioning have been reported (Britten et al., 2014; Rabin et al., 2014; Davis et al., 2015) and only a single dose of sequential mixed beam radiation was used to assess effects on operant conditioning in the current study. The age, strain, and sex of the animals, the radiation qualities of the radiation exposure(s), and the time interval between exposure and testing involved in a study may modulate the effects on measures of operant conditioning.

The interaction between simplified GCR exposure and HU appears to be complex and/or the effects of combined radiation and HU exposure attenuated compared with each environmental challenge alone. Although HU sham-irradiated animals showed impaired spatial habituation learning, there was a trend toward spatial habituation learning in animals following combined exposure to radiation and HU. Similarly, while increased depressive-like behavior was seen in irradiated mice it was not seen in animals following combined exposure to radiation and HU. Consistent with this patter, HU affected more metabolic pathways in plasma and brain of sham-irradiated than irradiated animals. Similarly, there was a directional reversal change in metabolite levels in animals following combined exposure to radiation and HU compared to metabolite levels in animals following exposure to only radiation or HU. Interestingly, enhanced dendritic spine formation following HU was reported in the sensorimotor cortex (Trinel et al., 2013) while simulated space radiation typically decreases dendritic spine formation in the hippocampus (Parihar and Limoli, 2013; Allen et al., 2015). We recognize that as we started the HU 5 days prior to the radiation exposure, the HU challenge might have served as a preconditioning challenge mitigating the effects of radiation. As HU continued 25 days after radiation exposure, it is also conceivable that the radiation exposure modulates the effects of HU following the radiation exposure. We recognize that astronauts will begin to experience the effects of microgravity prior to receiving the bulk of their GCR dose on any extended duration exploration class mission and that the GCR exposure will differ from the way it was administered herein. In space, the GCR exposure will be relative constant throughout the mission. This initial study involving WAG rats was performed to determine whether there were any interactive effects based on exposure to a simplified GCR spectrum delivered at once. Future efforts are warranted to determine whether the effects seen are limited to WAG rats or also seen in Fischer rats. The timing and duration of HU compared to the radiation exposure might be critical to consider in future studies. In analyzing the combined effects of traumatic brain injury and radiation, the sequence of the two challenges, time interval, seemed critical in determining the combined effect on the brain (Dicarlo et al., 2008; Allen et al., 2014; Dickstein et al., 2018).

In summary, the effects of HU and radiation on behavioral and cognitive performance and metabolic pathways in plasma and brain illustrate the complex interaction of environmental stressors pertinent to assessment and mitigation of risk to astronauts during and following space missions and highlights the importance of assessing these interactions. The metabolomics data suggest that it will be possible to develop stable plasma measures of the response to a simplified, simulated GCR exposure, HU, and to combined simGCRsim and HU exposure, and of behavioral and cognitive performance that could be used for developing and testing of mitigators. For acquiring converging evidence critical for CNS risk assessment during and following (deep) space missions, future efforts are warranted to assess the effects of HU and simulated space radiations in other strains.

## Data Availability Statement

The original contributions presented in the study are included in the article/Supplementary Material, further inquiries can be directed to the corresponding author.

## Ethics Statement

The animal study was reviewed and approved by IACUC OHSU.

## Author Contributions

JR and JB designed the study. SH, RS, BG, RH, ML, KR, NN, JS, RP, SL, JM, JC, JS, CO, and GB acquired and analyzed the data. SL, AB, AKw, and JW performed the HU procedures. All authors contributed to the review and editing of the manuscript.

## Conflict of Interest

The authors declare that the research was conducted in the absence of any commercial or financial relationships that could be construed as a potential conflict of interest.

## Publisher’s Note

All claims expressed in this article are solely those of the authors and do not necessarily represent those of their affiliated organizations, or those of the publisher, the editors and the reviewers. Any product that may be evaluated in this article, or claim that may be made by its manufacturer, is not guaranteed or endorsed by the publisher.
