# Supplementary material for: Effects of 5-Ion Beam Irradiation and Hindlimb Unloading on Metabolic Pathways in Plasma and Brain of Behaviorally Tested WAG/Rij Rats
Source: Front Physiol. 2021 Sep 27;12:746509. doi: 10.3389/fphys.2021.746509 (PMC8503608; doi:10.3389/fphys.2021.746509)
Supplement: Supplementary file 1 [file Data_Sheet_1.docx]

**Supplementary figure legends**

**Suppl. Fig. 1. A.** No preferential exploring of the novel object in sham-irradiated or irradiated rats. **B.** No preferential exploring of the novel object in sham-irradiated and irradiated animals in the absence and presence of HU.

**Suppl. Fig. 2. A.** No group difference sin body weights prior to frontal cortical cognitive testing. **B.** No group difference in visual cue learning (errors to criterion). **C.** No group differences in 24 hr recall in visual cue learning. **D.** No group differences in during set shift errors (errors to criterion. **E.** No group difference in 24 hr recall during set shift errors. Set shifting was not impaired in any group. **F.** No group differences in perseverative errors. **G.** No group differences in regressive errors. **H.** No group differences in never reinforced errors.

**Suppl. Fig. 3. A.** No group differences in reversal errors (errors to criterion). **B.** No group differences in perseverative errors. **C.** No group differences in regressive errors. **D.** No group differences in motivation to obtain food.

**Suppl. Fig. 4.** There were no group differences in the number of sessions to criterion for single lever trainings (**A**), in the number of sessions to criterion for retract lever training (**B**), in the total number of pre-training sessions to reach criterion (**C**).

**Suppl. Fig. 5.** There were no group differences in cumulative active lever presses for the duration of the progressive ratio session.

**Suppl. Fig. 6.** No group differences in body weights prior to anesthesia.

**Suppl. Fig. 7. A.** Multiple metabolites in plasma and cortex in affected pathways in the four groups: purine metabolism, taurine and hypotaurine metabolism, phenylalanine, tyrosine, and tryptophan biosynthesis, phenylalanine metabolism, and arginine and proline metabolism. Arrows indicate metabolite conversions within each simplified metabolic pathway, and each box and whisker plot shows metabolite concentrations based on peak value. **B.** Multiple metabolites in the cortex related to arginine and proline metabolism were significantly altered by radiation and/or HU treatment. Arrows indicate metabolite conversions within each simplified metabolic pathway, and each box and whisker plot shows metabolite concentrations based on peak value. The outliers identified by the software in each group are indicated by the distinct symbols above or below the box and whisker plots.

**Suppl. Fig. 8. A.** Multiple metabolites in the hippocampus in affected pathways in the four groups: purine metabolism, taurine and hypotaurine metabolism, phenylalanine, tyrosine, and tryptophan biosynthesis, phenylalanine metabolism, and arginine and proline metabolism based on the raw data. Arrows indicate metabolite conversions within each simplified metabolic pathway, and each box and whisker plot shows metabolite concentrations based on peak value. **B.** Multiple metabolites in the hippocampus related to purine metabolism, taurine and hypotaurine metabolism, phenylalanine, tyrosine, and tryptophan biosynthesis, phenylalanine metabolism, and arginine and proline metabolism based on the normalized data. Arrows indicate metabolite conversions within each simplified metabolic pathway, and each box and whisker plot shows metabolite concentrations based on peak values normalized to tissue weight. The outliers identified by the software in each group are indicated by the distinct symbols above or below the box and whisker plots.
