# Supplementary figures and images for: Effects of 5-Ion Beam Irradiation and Hindlimb Unloading on Metabolic Pathways in Plasma and Brain of Behaviorally Tested WAG/Rij Rats
Source: Front Physiol. 2021 Sep 27;12:746509. doi: 10.3389/fphys.2021.746509 (PMC8503608; doi:10.3389/fphys.2021.746509)

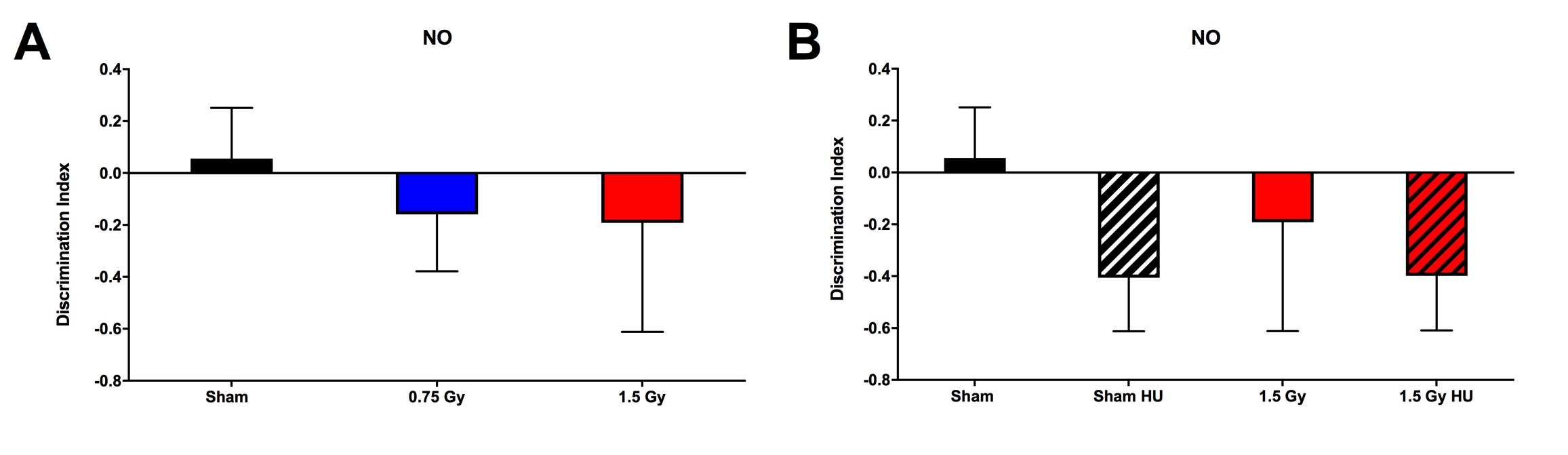

Supplement: Supplementary file 2 [file Image_1.JPEG]

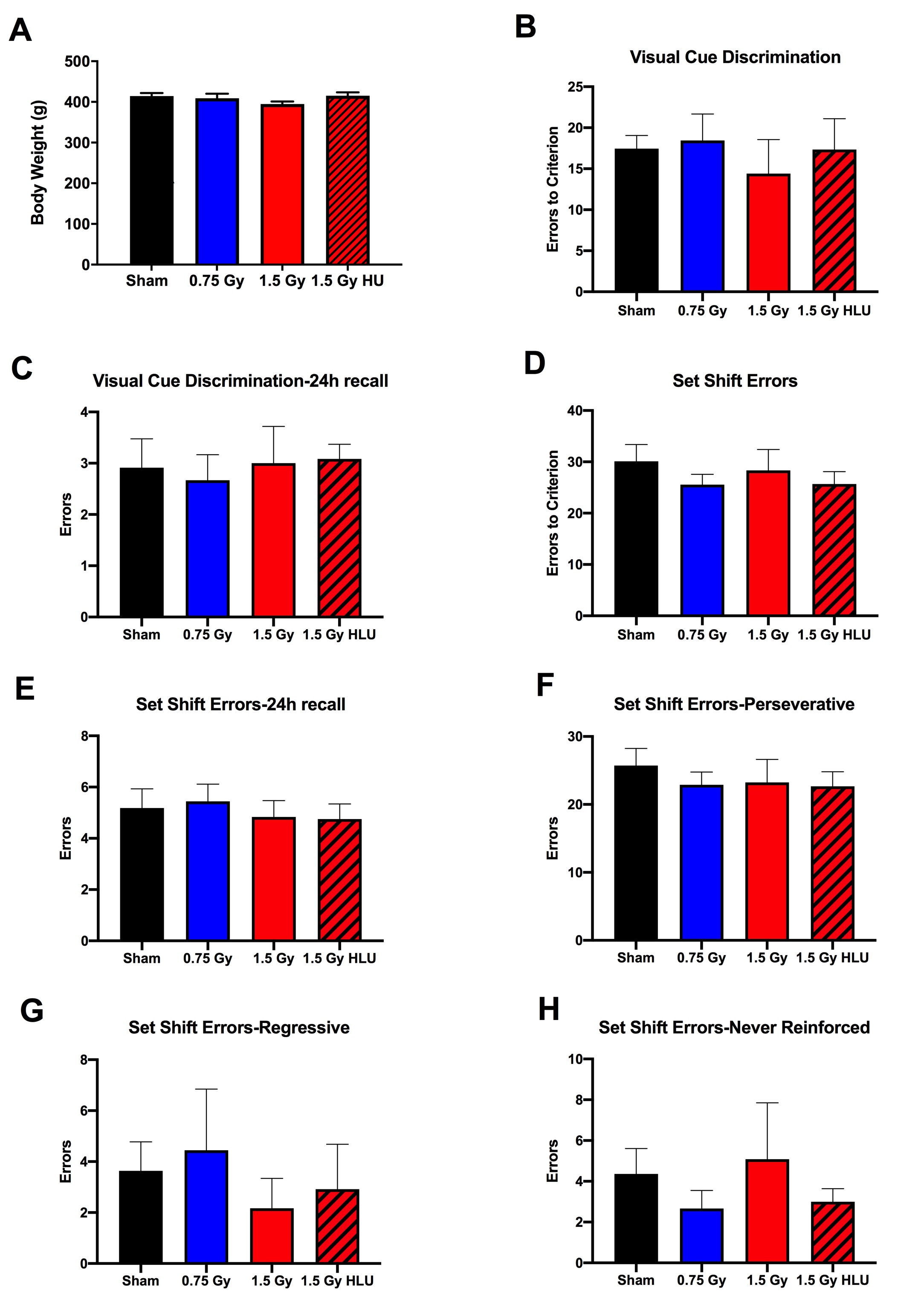

Supplement: Supplementary file 3 [file Image_2.JPEG]

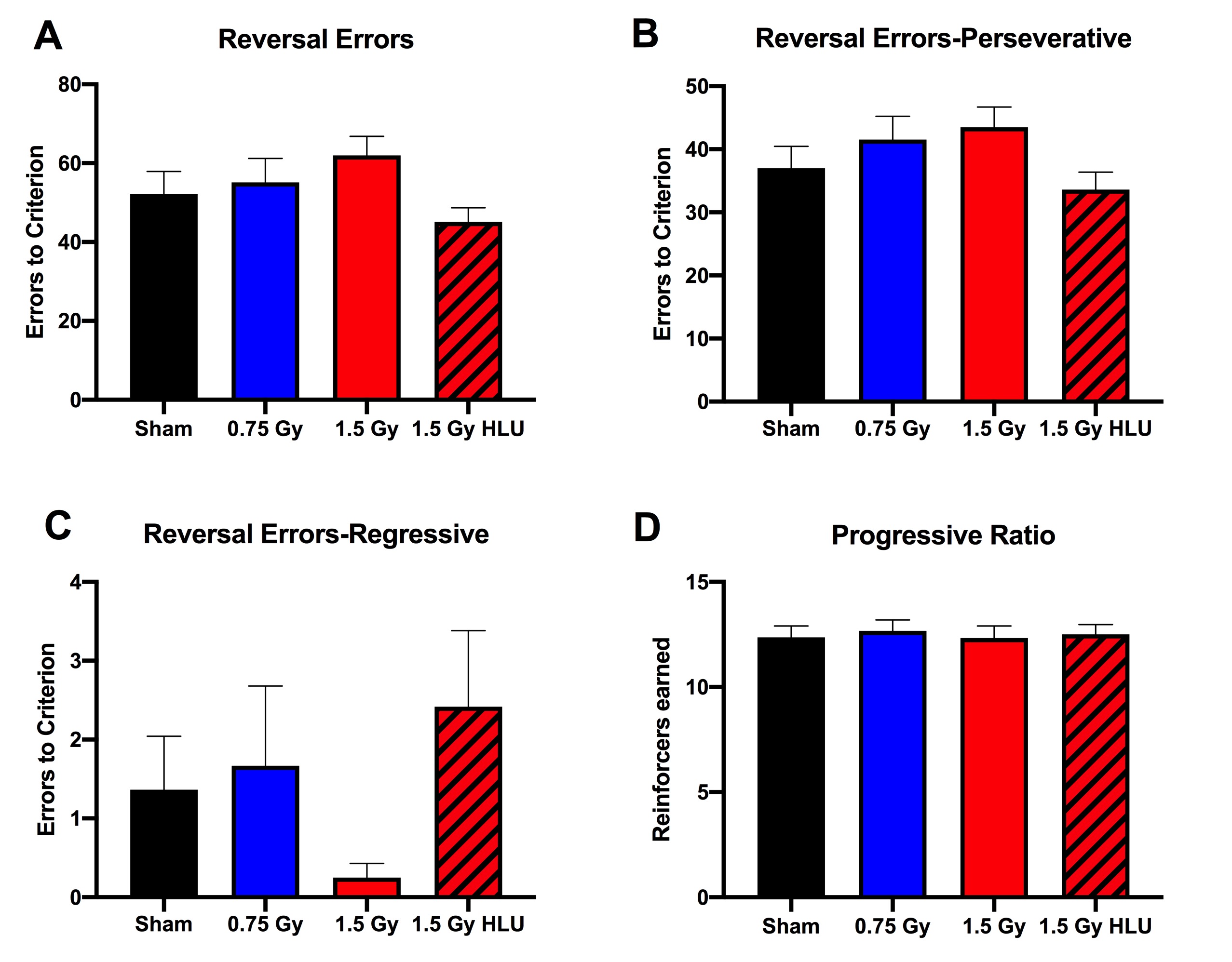

Supplement: Supplementary file 4 [file Image_3.JPEG]

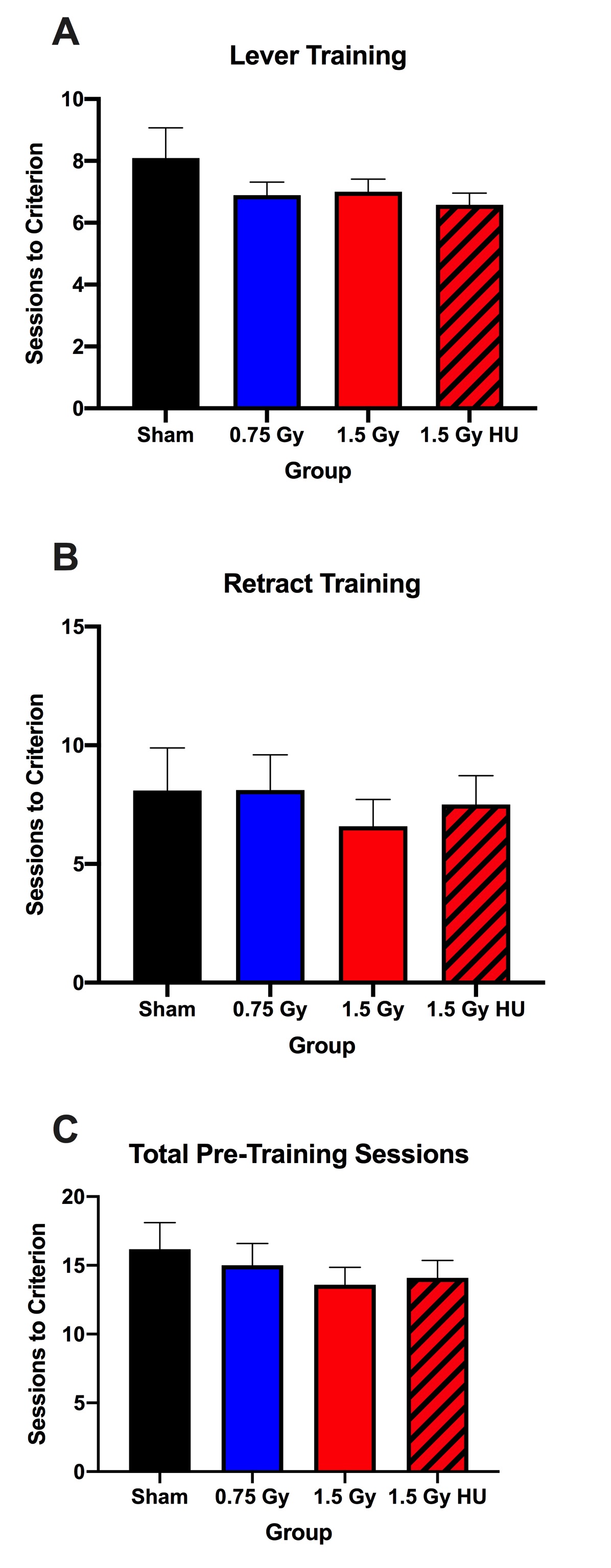

Supplement: Supplementary file 5 [file Image_4.JPEG]

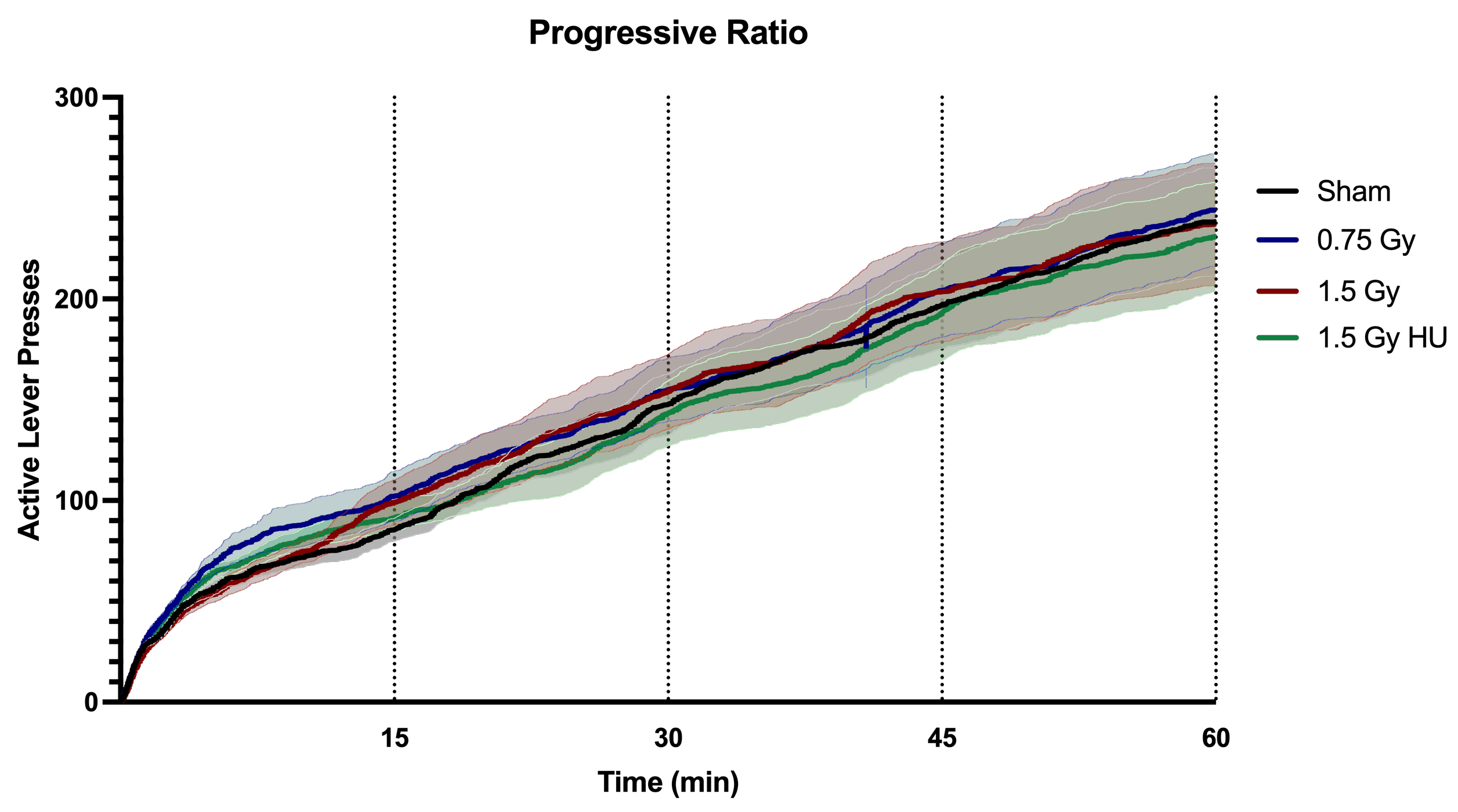

Supplement: Supplementary file 6 [file Image_5.PNG]

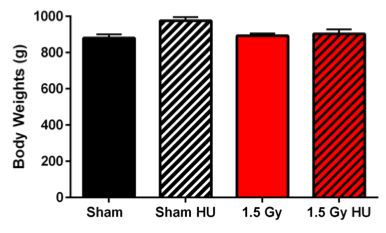

Supplement: Supplementary file 7 [file Image_6.TIFF]

A

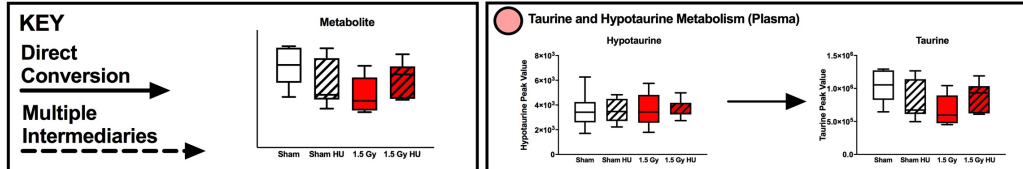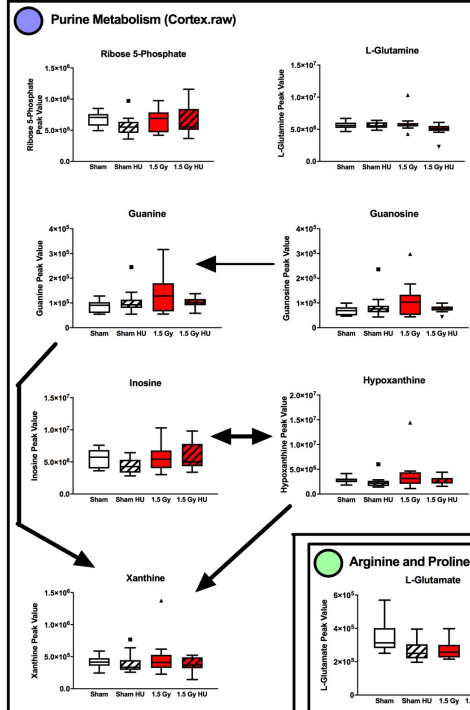

## PATHWAYS

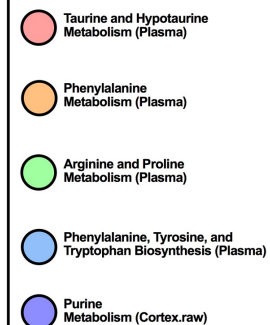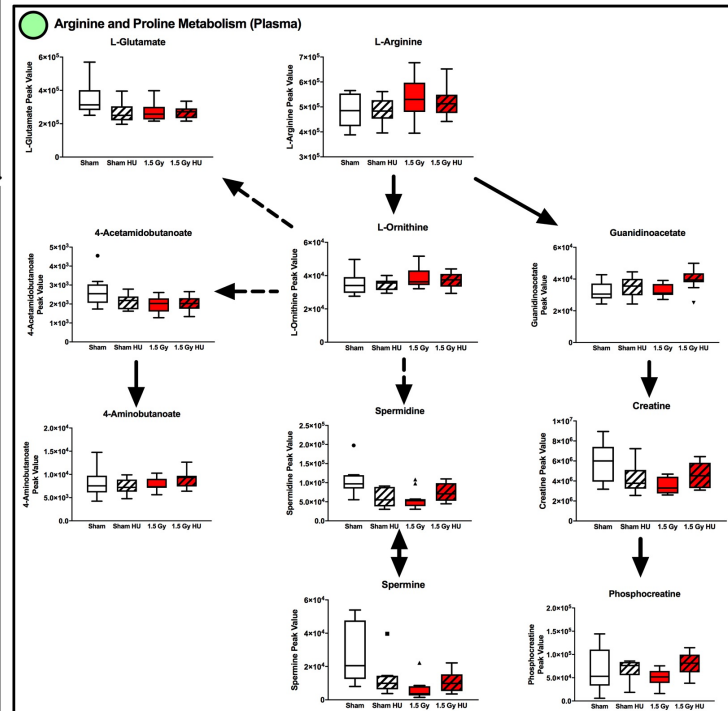

B

## KEY

Direct Conversion →  
Multiple Intermediaries →

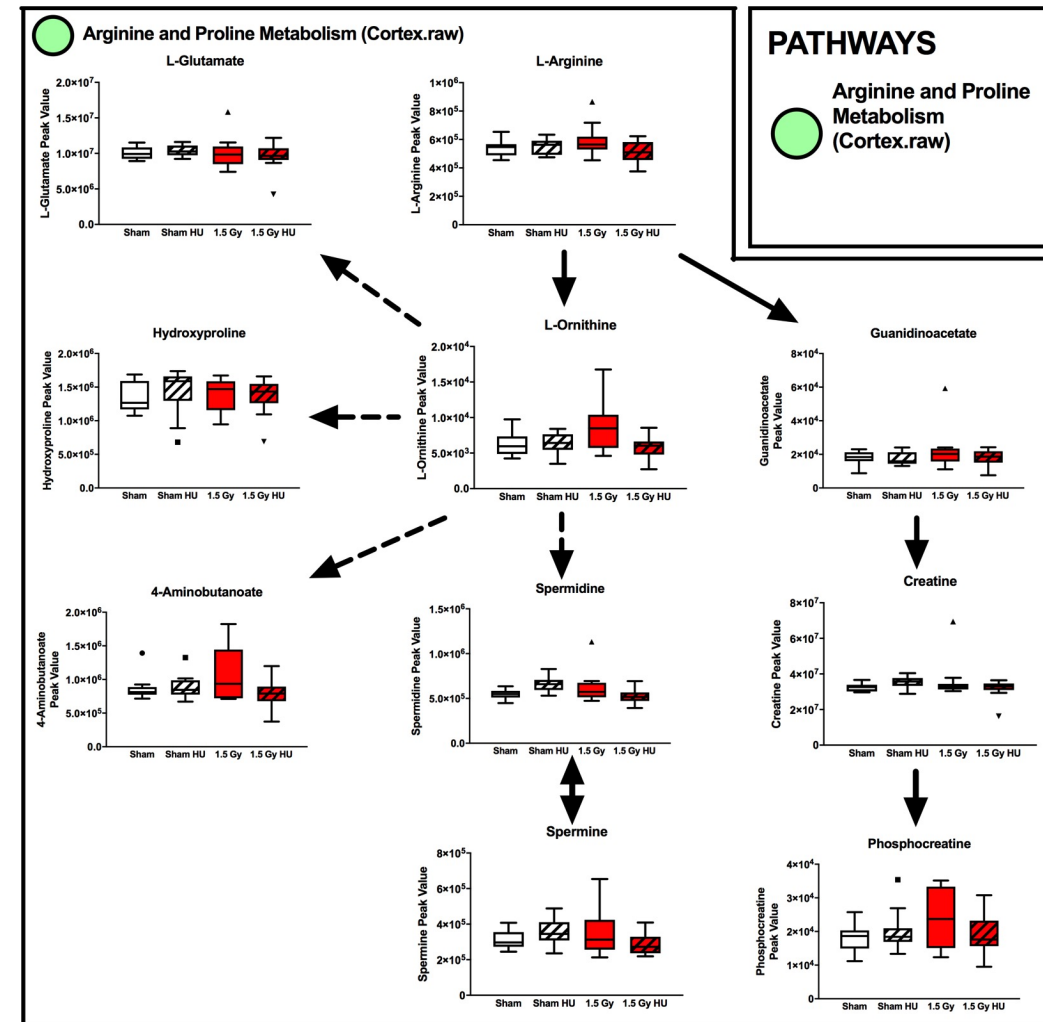

Supplement: Supplementary file 8 [file Image_7.pdf]

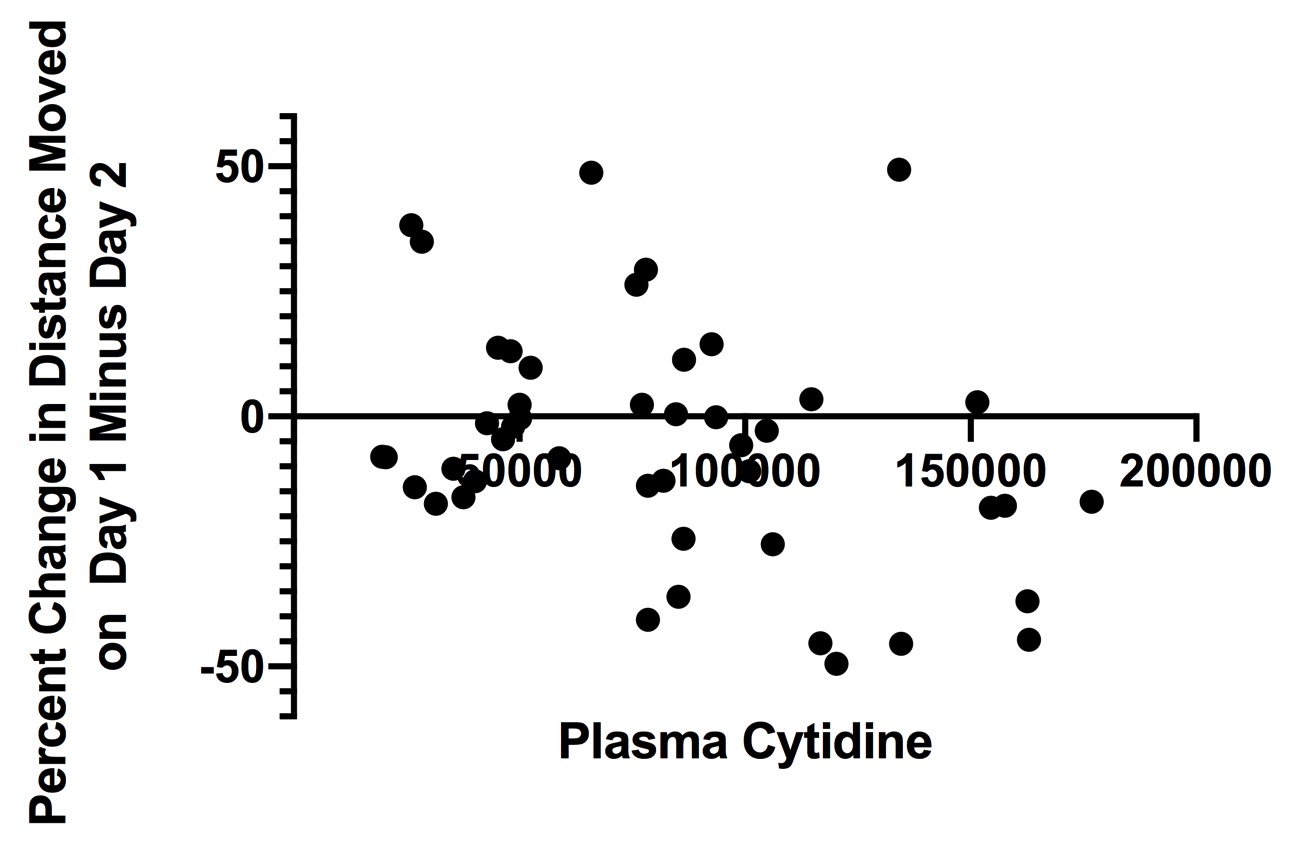

Supplement: Supplementary file 10 [file Image_9.jpg]
