# Supplementary material for: Effects of 5-Ion Beam Irradiation and Hindlimb Unloading on Metabolic Pathways in Plasma and Brain of Behaviorally Tested WAG/Rij Rats
Source: Front Physiol. 2021 Sep 27;12:746509. doi: 10.3389/fphys.2021.746509 (PMC8503608; doi:10.3389/fphys.2021.746509)
Supplement: Supplementary file 9 [file Image_8.pdf]

A

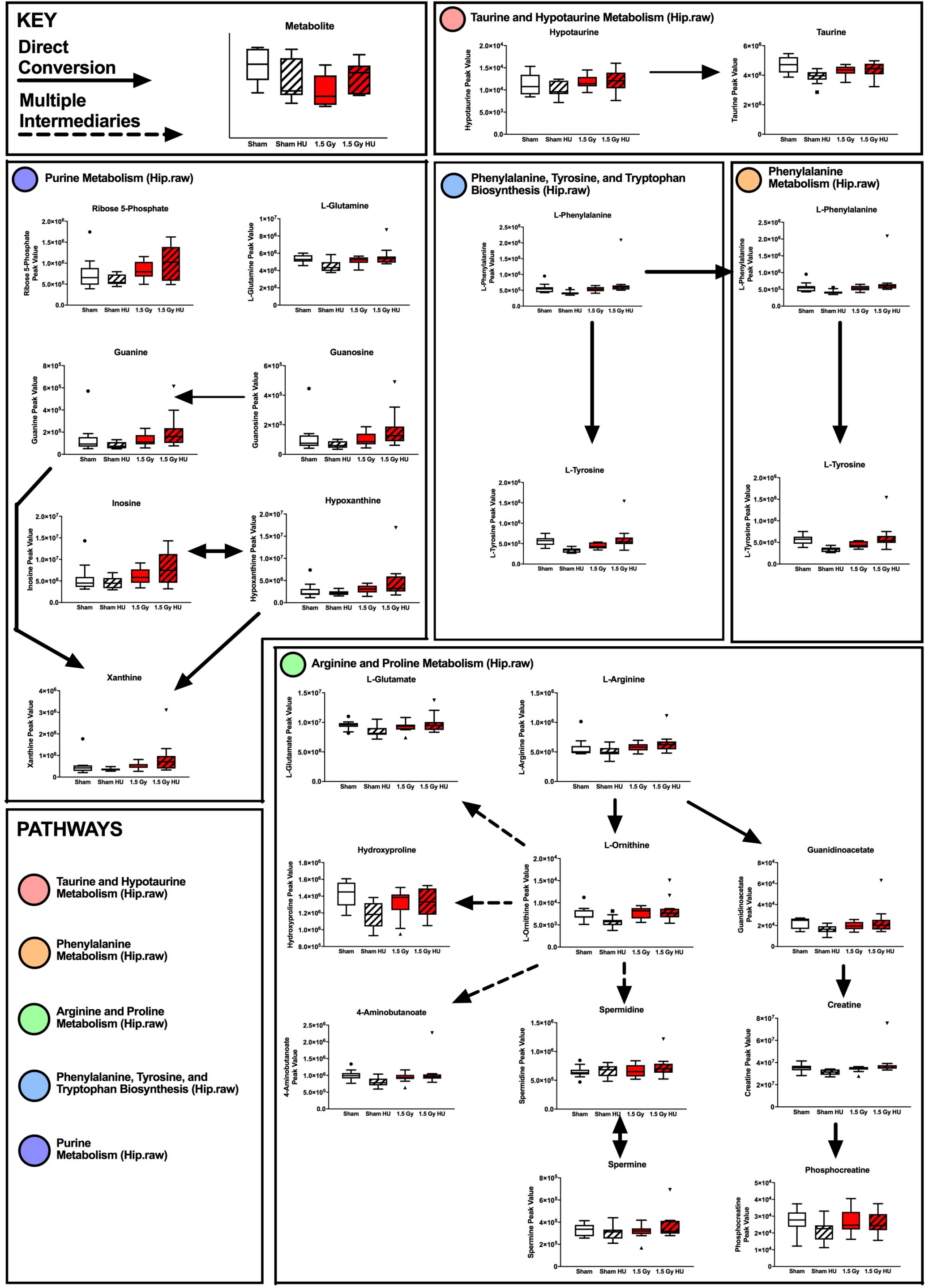
KEY
Direct Conversion
Multiple Intermediaries
Metabolite
Sham
Sham HU
1.5 Gy
1.5 Gy HU
Taurine and Hypotaurine Metabolism (Hip.raw)
Hypotaurine
Taurine
Purine Metabolism (Hip.raw)
Ribose 5-Phosphate
L-Glutamine
Guanine
Guanosine
Inosine
Hypoxanthine
Xanthine
Phenylalanine, Tyrosine, and Tryptophan Biosynthesis (Hip.raw)
L-Phenylalanine
L-Tyrosine
Phenylalanine Metabolism (Hip.raw)
Arginine and Proline Metabolism (Hip.raw)
L-Glutamate
L-Arginine
L-Ornithine
Hydroxyproline
Guanidinoacetate
4-Aminobutanoate
Spermidine
Creatine
Spermine
Phosphocreatine
PATHWAYS
Taurine and Hypotaurine Metabolism (Hip.raw)
Phenylalanine Metabolism (Hip.raw)
Arginine and Proline Metabolism (Hip.raw)
Phenylalanine, Tyrosine, and Tryptophan Biosynthesis (Hip.raw)
Purine Metabolism (Hip.raw)

B

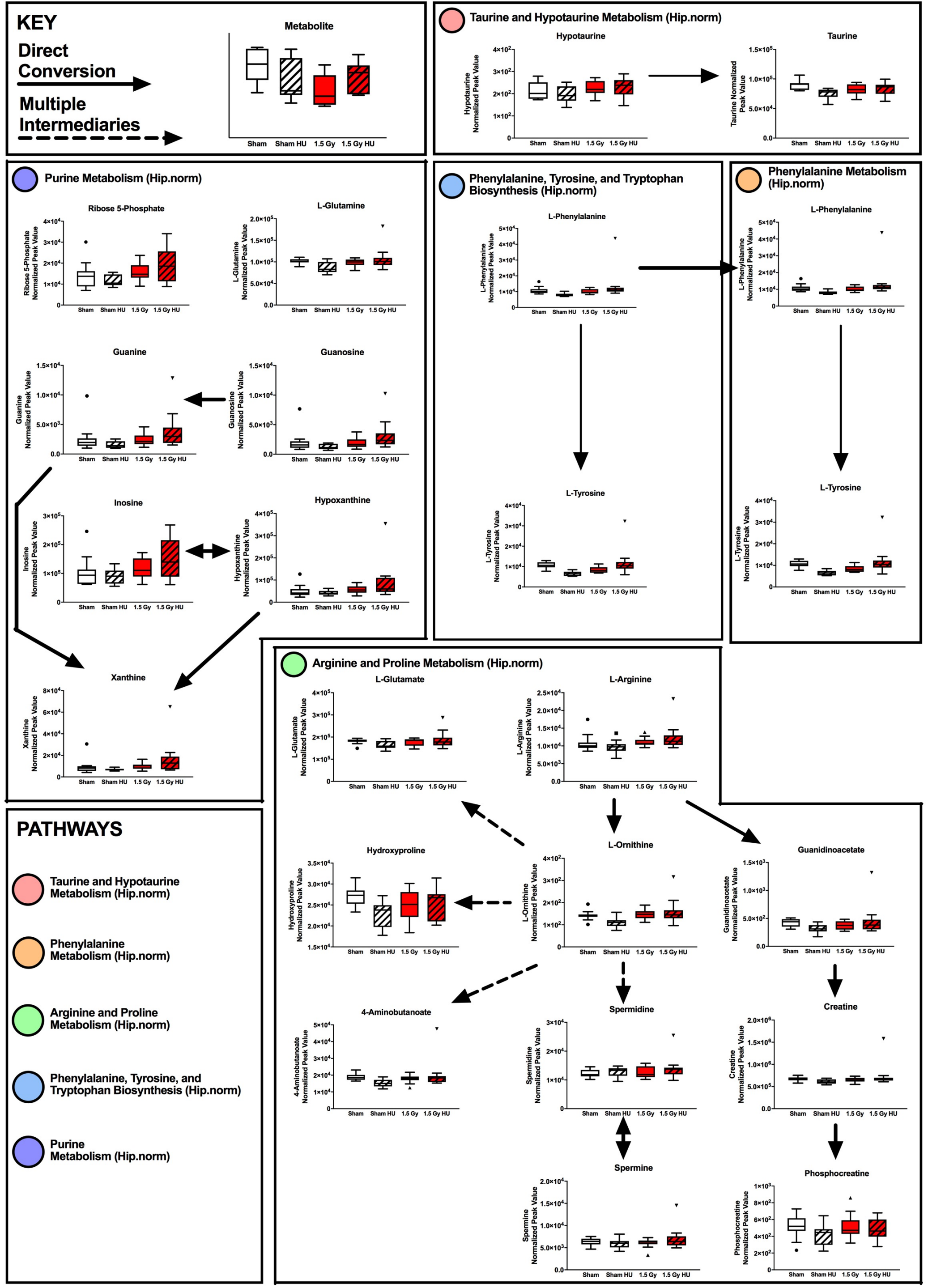
KEY
Direct Conversion
Multiple Intermediaries
Metabolite
Sham
Sham HU
1.5 Gy
1.5 Gy HU
Taurine and Hypotaurine Metabolism (Hip.norm)
Hypotaurine
Taurine
Purine Metabolism (Hip.norm)
Ribose 5-Phosphate
L-Glutamine
Guanine
Guanosine
Inosine
Hypoxanthine
Xanthine
Phenylalanine, Tyrosine, and Tryptophan Biosynthesis (Hip.norm)
L-Phenylalanine
L-Tyrosine
Phenylalanine Metabolism (Hip.norm)
Arginine and Proline Metabolism (Hip.norm)
L-Glutamate
L-Arginine
L-Ornithine
Hydroxyproline
Guanidinoacetate
4-Aminobutanoate
Spermidine
Creatine
Spermine
Phosphocreatine
PATHWAYS
Taurine and Hypotaurine Metabolism (Hip.norm)
Phenylalanine Metabolism (Hip.norm)
Arginine and Proline Metabolism (Hip.norm)
Phenylalanine, Tyrosine, and Tryptophan Biosynthesis (Hip.norm)
Purine Metabolism (Hip.norm)
